# The PP2A/4/6 subfamily of phosphoprotein phosphatases regulates DAF-16 and confers resistance to environmental stress in postreproductive adult *C*. *elegans*

**DOI:** 10.1371/journal.pone.0229812

**Published:** 2020-12-14

**Authors:** Rebecca S. Rivard, Julia M. Morris, Matthew J. Youngman

**Affiliations:** Department of Biology, Villanova University, Villanova, PA, United States of America; George Washington University, UNITED STATES

## Abstract

Insulin and insulin-like growth factors are longevity determinants that negatively regulate Forkhead box class O (FoxO) transcription factors. In *C*. *elegans* mutations that constitutively activate DAF-16, the ortholog of mammalian FoxO3a, extend lifespan by two-fold. While environmental insults induce DAF-16 activity in younger animals, it also becomes activated in an age-dependent manner in the absence of stress, modulating gene expression well into late adulthood. The mechanism by which DAF-16 activity is regulated during aging has not been defined. Since phosphorylation of DAF-16 generally leads to its inhibition, we asked whether phosphatases might be necessary for its increased transcriptional activity in adult *C*. *elegans*. We focused on the PP2A/4/6 subfamily of phosphoprotein phosphatases, members of which had been implicated to regulate DAF-16 under low insulin signaling conditions but had not been investigated during aging in wildtype animals. Using reverse genetics, we functionally characterized all *C*. *elegans* orthologs of human catalytic, regulatory, and scaffolding subunits of PP2A/4/6 holoenzymes in postreproductive adults. We found that PP2A complex constituents PAA-1 and PPTR-1 regulate DAF-16 transcriptional activity during aging and that they cooperate with the catalytic subunit LET-92 to protect adult animals from ultraviolet radiation. PP4 complex members PPH-4.1/4.2, and SMK-1 also appear to regulate DAF-16 in an age-dependent manner, and together with PPFR-2 they contribute to innate immunity. Interestingly, SUR-6 but no other subunit of the PP2A complex was necessary for the survival of pathogen-infected animals. Finally, we found that PP6 complex constituents PPH-6 and SAPS-1 contribute to host defense during aging, apparently without affecting DAF-16 transcriptional activity. Our studies indicate that a set of PP2A/4/6 complexes protect adult *C*. *elegans* from environmental stress, thus preserving healthspan. Therefore, along with their functions in cell division and development, the PP2A/4/6 phosphatases also appear to play critical roles later in life.

## Introduction

The Insulin and IGF-signaling (IIS) pathway is a primary modulator of lifespan across evolutionarily diverse species [1]. Genetic and behavioral changes that modify IIS pathway activity change the quality and length of life dramatically. Loss-of-function mutations in the insulin receptor correlate with a 2- to 3-fold extension in the lifespan in some species [2]. Moreover, insulin signaling mutants appear more youthful and are more resistant to a variety of environmental insults, demonstrating a connection between stress resistance and lifespan [3, 4]. How well an organism contends with stressful stimuli is a gauge of the potency of its maintenance and repair systems, which, together with its defense strategies, constitute the whole of its means of evading or counteracting the damage that contributes to age-related decline. Since the IIS pathway and its ultimate target, the Forkhead box family (FoxO) transcription factor, profoundly influence lifespan and stress resistance, understanding how they are regulated over time could suggest the basis for the milestones of aging and reveal potential avenues for manipulating the rate of aging.

In the roundworm *Caenorhabditis elegans* the FoxO protein DAF-16 is a transcriptional regulator that determines lifespan in part by conferring resistance to environmental insults. The transcriptional activity of DAF-16 is inhibited when insulin-like ligands bind to the insulin receptor, DAF-2, triggering the kinase AKT-1 to modify DAF-16 with an inhibitory phosphate group that excludes it from the nucleus. In the absence of these soluble ligands, DAF-16 translocates to the nucleus and upregulates the expression of genes that promote longevity, and it is because of this function of DAF-16 that *daf-2* mutants live longer than their wildtype counterparts [5]. That is, whereas wildtype worms achieve reproductive maturity three days after hatching from eggs and live for about another 20 days as adults, the adult portion of a *daf-2* mutant’s life may stretch for more than 50 days. Even if the IIS pathway is intact DAF-16 activity may be triggered when animals encounter harmful stimuli including high temperature and ultraviolet radiation [6]. Under these stress-inducing conditions, DAF-16 targets may include genes that encode detoxifying enzymes, immune effectors, and chaperones that restore homeostasis, preserve cellular health, and contribute to host defense [7]. Recently we and others have shown that DAF-16 is activated in an age-dependent manner, despite the absence of acute stress. During adulthood, the expression levels of the *daf-16a* splice isoform, and to an even greater extent the *daf-16d* isoform progressively increase [8]. This is coupled to increased expression of a subset of DAF-16 transcriptional targets in adults as compared to in larvae, indicating that DAF-16 is activated as *C*. *elegans* age [9, 10].

One functional consequence of its increased activity during adulthood is an age-dependent requirement for DAF-16 in innate immunity. When DAF-16 is inhibited specifically in adult animals even before they encounter a pathogen, their ability to resist a bacterial infection is compromised [10]. This suggests that the immune protection conferred by DAF-16 during adulthood is associated with the intrinsic sustained increase in its transcriptional activity that begins during reproduction and persists into late adulthood. Consistent with this possibility, infection does not seem to induce DAF-16 activation in adult *C*. *elegans* [10, 11].

In a previous study we asked about the regulatory mechanism responsible for activating DAF-16 in adult animals, and we found the nuclear protein SMK-1 to be required for the age-dependent increase in expression of DAF-16 transcriptional targets [10]. Similar to DAF-16, SMK-1 also contributes to innate immunity in post-reproductive adult *C*. *elegans*. Prior to our studies, *smk-1* had been characterized in two contexts: *daf-2* mutants and during development in wild type animals. In *daf-2(e1370)* mutants when DAF-16 is constitutively de-repressed *smk-1* is required for both lifespan extension and stress resistance [12]. Without SMK-1, the expression of a subset of DAF-16 transcriptional targets in *daf-2(e1370)* mutants is reduced because of a deficiency in transcription initiation [13]. To regulate transcription in *daf-2(e1370)* animals, SMK-1 functions together with PPH-4.1, the catalytic subunit of the phosphoprotein phosphatase 4 (PP4) complex in *C*. *elegans*. Interestingly, SMK-1 also cooperates with PPH-4.1 during embryonic development in *C*. *elegans*, to silence the CHK-1-mediated response to DNA damage on replicating chromatin and thus allow cell cycle progression [14]. SMK-1 is orthologous to *D*. *melanogaster* flfl, *S*. *cerevisiae* Psy2p, and human PP4R3, a regulatory subunit of the PP4 complex. Whether a PPH-4.1/SMK-1 complex functions during adulthood in wildtype *C*. *elegans* is not known.

PP4 is part of a subfamily of phosphoprotein phosphatases that also includes PP2A and PP6. Phosphoprotein phosphatases (PPPs) comprise a large class of evolutionarily conserved enzymes, that antagonize kinases by removing phosphate groups from a broad repertoire of substrates. In evolutionarily diverse species, the PP2A/4/6 subfamily regulates a wide array of processes, including cell cycle progression, meiosis, cellular differentiation, and the activity of multiple signaling pathways including the IIS pathway [15–25]. In *C*. *elegans* PP4 is not the only member of the PP2A/4/6 family with a connection to the insulin signaling pathway. Just as the PP4 subunit SMK-1 is required for the extended lifespan of *daf-2* mutants, so too is the regulatory subunit of the PP2A complex PPTR-1 [12]. In mammals, the inhibitory phosphorylation imparted to FoxO transcription factors by AKT is opposed by the action of phosphoprotein phosphatase PP2A. Both FoxO1 and FoxO3a are substrates of PP2A, and biochemical evidence indicates that PP2A selectively dephosphorylates the AKT phosphorylation sites of FoxO family members [26–29]. Moreover, PP2A also dephosphorylates AKT itself, thereby inactivating it [30–32]. This particular mechanism of PP2A functioning as an indirect positive regulator of FoxO transcription factors appears to be conserved in *C*. *elegans*. In *daf-2* mutants, the constitutive activation of DAF-16 requires PPTR-1, a conserved regulatory subunit of PP2A complexes [33]. PPTR-1 associates with AKT-1 but not DAF-16 in worms, and overexpression of PPTR-1 reduces the levels of phosho-AKT-1. Since as a whole the expression levels of insulin-like peptides considered to be agonists of DAF-2 either remain constant or increase during adulthood, the presence of a sustained inhibitory signal through the IIS pathway that persists in adult animals seems likely [9]. A regulator that can counteract this signal is therefore an attractive candidate for functioning to modulate the activity of DAF-16 during aging, and this raises the possibility that multiple members of the PP2A/4/6 family may contribute to the age-dependent functions of DAF-16.

PPPs can be divided into two evolutionarily distinct branches composed of PP1/PP2A/PP2B and PP5/PPEF/PP7 based on divergent regulatory and catalytic domains, with the PP2A subgroup containing the PP2A/PP4/PP6 subfamily [34]. The PP1/2A/4/6 catalytic subunits all have a highly conserved globular domain that acts in concert with one (PP1/6) or two (PP2A/4/6) binding partners. Catalytic subunits of the PP2A/4/6 subfamily associate with regulatory proteins and, in some cases, a scaffolding protein to comprise a functional holocomplex. Multiple versions of the PP2A/4/6 complexes are possible because there are several genes whose products bind and regulate the same catalytic subunit [35]. For example, in humans there are 18 different regulatory proteins capable of associating with the PP2A enzyme, allowing for the generation of nearly 100 holoenzymes [15, 36]. The combinatorial diversity of these complexes is believed to contribute to the subcellular localization, substrate specificity, and thus the functional capacity of the phosphoprotein phosphatases, depending on their subunit composition [37]. Considering that different combinations of subunits allow for the possibility of multiple versions of the PP2A/4/6 complexes and in light of the apparent functional overlap in PP2A and PP4 in regulating DAF-16 in a genetic background where it is hyper-activated, we sought to identify the specific individual constituents of the PPP complexes that might be required for the age-dependent function of DAF-16.

Here we present the first systematic investigation of the role of the PP2A/4/6 subfamily of phosphoprotein phosphatases during aging in wildtype *C*. *elegans*. We used a reverse genetic approach to functionally characterize all *C*. *elegans* orthologs of human PP2A/4/6 catalytic, scaffold, and regulatory subunits in juvenile and postreproductive adult animals. Specifically, we used RNAi to individually knock down the genes encoding these proteins and examined the effect on the ability of adult *C*. *elegans* to resist bacterial infection, ultraviolet irradiation, and thermal stress. Our data suggest that the PP2A and PP4 complexes are important for the age-dependent activity of DAF-16. Moreover, we present evidence to support the existence of multiple versions of PP2A, PP4, and PP6 complexes with apparent specialized functions to protect adult *C*. *elegans* from different environmental stresses and thus as a group contribute to preserving healthspan.

## Methods

### *In silico* analyses of *C. elegans* PP2A/4/6 catalytic subunits

To compare the PP2A/4/6 catalytic subunits of the *C*. *elegans* to their human orthologs, amino acid sequences of LET-92, PPH-4.1, PPH-4.2 and PPH-6 were downloaded from Wormbase (wormbase.org) and the amino acid sequences of PPP2cA, PPP4c, and PPP6c were downloaded from Genbank (ncbi.nlm.nih.gov/genbank). The sequences were then aligned using Clustal Omega multiple sequence alignment tool (ebi.ac.uk/msa/clustalo/), and conserved residues and motifs were manually annotated based on existing alignments of phosphoprotein phosphatase (PPP) proteins [38–40]. Information depicted in the alignment was consolidated in a sequence logo created using weblogo (weblogo.threeplusone.com). To preserve the alignment of sequences within the logo, internal gaps incorporated during the Clustal alignment were made to be represented by “X” instead of “-“. Then, since weblogo requires all the input sequences to be the same length, each sequence was truncated from the N-terminus to match the length of the shortest of the seven proteins, PPP6c (305 amino acids). This corresponds to a range of between 1 (for PPP4c) and 26 N-terminal amino acids (for PPH-4.1 and PPH-6) that were excluded from the sequence logo. Conserved residues and motifs were manually annotated as described above.

### *C. elegans* growth and maintenance

Bristol N2 and VIL001 *mjyIs001 [Plys7*::*gfp] C*. *elegans* strains were grown and maintained under standard laboratory conditions [41]. To generate strain VIL001, stable transgenic animals harboring an extrachromosomal *lys-7* promoter::gfp fusion construct [42] were subjected to gamma irradiation to yield a chromosomal integration of *Plys7*::*gfp*. F_2_ segregants of the irradiated P_0_ animals yielding 100% GFP-expressing progeny were selected for further analysis. One of these lines was backcrossed to the N2 wildtype strain seven times and then designated VIL001.

### *C. elegans* synchronization

Worms were synchronized via sodium hypochlorite treatment [43]. Briefly, gravid adult hermaphrodites were treated with a solution of bleach and KOH to release eggs which were recovered by centrifugation and washed several times in sterile M9 buffer (41 mM Na_2_HPO_4_, 22 mM KH_2_PO_4_, 8.6 mM NaCl, 19 mM NH_4_Cl). After hatching overnight in M9 buffer, approximately 2000 first larval stage L1 worms were dropped to fresh, pre-seeded RNAi plates or to Nematode Growth Medium (NGM; 3 g/L NaCl, 25 g/L agar, 2.5 g/L peptone, 5 mg/L cholesterol, 1mM MgSO_4_, 1mM CaCl_2_, 2.5% (v/v) KPO_4_ buffer, pH 6) plates seeded with *E*. *coli* OP50.

### RNAi treatment

Animals were treated with RNAi through their food source as described by Fraser *et al*. with modifications as described below [44]. All RNAi sequences were confirmed by sequencing prior to use. Bacteria from the Source BioScience library were streaked onto LB plates containing ampicillin (amp, 50μg/mL) and tetracycline (tet, 1.25μg/mL) and allowed to grow overnight at 37°C. One colony from each plate was used to inoculate 200mL of LB with amp (1ng/mL) and grown overnight in a 37°C shaker. Bacteria were centrifuged at 5,000g for 10 minutes, resuspended in 20mL of LB containing amp, and 1mL was dropped onto individual RNAi plates (standard NGM plates with 1mg/mL carbenicillin, 2μM IPTG). Lawns of *E*. *coli* RNAi clones were allowed to grow for at least two days before worms were introduced to the plates. Except in the cases where either *let-92* or *paa-1* were the targets of the treatment, synchronized cohorts of L1 larvae were dropped onto seeded RNAi plates and maintained at 20°C. When these animals reached the fourth larval stage (L4) they were transferred to pre-seeded RNAi plates containing 25 μg/mL FUdR (5-fluorodeoxyuridine). Since initiating knockdown of *let-92* and *paa-1* at L1 prevented maturation past L4, RNAi treatment targeting these genes was delayed until animals had reached L4 on NGM plates seeded with OP50. Worms subjected to heat stress or UV irradiation remained on RNAi plates until they died. For bacterial infection assays, worms were transferred from the RNAi plates at either the L4 stage or at Day 6 of adulthood to plates containing *Pseudomonas aeruginosa* where they remained throughout the duration of the assay.

### *Pseudomonas aeruginosa* (PA14) infection assays

Infection assays were carried out as described in Tan *et al*. with the following modifications [45]. For each infection assay replicate worms were cultured on RNAi plates as described above. At L4 and Day 6 at least 30 animals were transferred to each of three slow kill assay 3.5 cm plates containing 25 μg/mL FUdR. These plates had been seeded with 6 μl of an overnight culture of *P*. *aeruginosa* strain PA14, incubated for one night at 37°C and then incubated a second night at room temperature prior to the start of the infection assay. Infections of wildtype N2 animals were carried out at 25˚C, and infections of *daf-16(mgDf47)* mutants were performed at 20˚C. Worm survival was scored in the morning and evening every day by counting the number of animals that responded to gentle prodding with a wire pick. Worms that failed to move were scored as dead and removed from the plates at each time point.

### Thermotolerance assay

After being synchronized by sodium hypochlorite treatment as described above, L1 larvae were dropped on to RNAi plates seeded with a clone expressing dsRNA to target a given gene of interest. Worms were maintained on those plates until the L4 larval stage when they were split into two groups. The first group of worms was subjected to thermal stress as described previously with some modifications [12]. Briefly, approximately 100 L4 worms were distributed evenly between three pre-seeded 3.5 cm RNAi food plates containing FUdR and then incubated at 35°C for the duration of the heat treatment and scored every few hours for survival with animals being counted as alive if they responded to gentle prodding with a wire pick. The second group of worms, consisting of ~2000 animals, was divided among three 6 cm pre-seeded RNAi + FUdR plates by chunking and were maintained at 20˚C. At the sixth day of adulthood, ~100 of these animals were subjected to heat stress according to the same procedure as outlined above for L4 thermal stress assays.

### UV irradiation assay

Synchronized groups of worms were cultured on RNAi plates beginning at the L1 larval stage. Worms were maintained on those plates until the L4 larval stage when they were split into two groups. The first group of approximately 100 worms was distributed among three 3.5 cm pre-seeded RNAi + FUdR plates for UV treatment. Plates were placed in an irradiator (UV Stratalinker® 1800, Stratagene) with lids removed and were irradiated with 1000 J/m^2^ UV. Following treatment worms were maintained at 20°C and scored for survival daily until all animals had died. Worms were scored as alive if they responded to gentle prodding with a wire pick. The remaining second group of L4 larval worms were transferred to RNAi + FUdR plates by chunking and were maintained at 20°C until they reached Day 6 of adulthood when they were subjected to the same UV treatment and scoring regimen.

### Fluorescence microscopy

Images of *Plys-7*::*GFP* expression in *C*. *elegans* were taken at Day 6 on a Nikon Eclipse E800 equipped with a Jenoptik digital camera. Worms were immobilized on agar pads using polybead® polystyrene 0.10μm microspheres (Polysciences, Inc.), and pictures were taken quickly thereafter to avoid undo stress. Transgenic animals expressing *Plys7*::*gfp* were assigned to one of three categories based on the relative GFP expression levels (low, medium, and high) according to the following criteria: low—overall weak expression of GFP in the intestine or GFP expression limited to anterior of intestine, just behind the pharynx; medium—bright GFP expression from the anterior intestine to the midbody but little to no expression in posterior intestine; high—robust GFP expression throughout the entire length of intestine. The percentage GFP expression for each sample was calculated using a minimum of 200 animals and then the percentages were averaged across at least 3 biological replicates.

### Statistical analyses

To calculate median lifespans (LT_50_s) a three parameter sigmoidal curve was fit to plots of survival (fraction of worms alive versus time) according to the general equation y = a/(1+e^(-(x-x0)/b)^) using SigmaPlot version 14 (Systat Software, San Jose, CA). This equation was used to determine the point at which 50% of the animals in the assay had died. The average fold difference between the LT_50_ of mutant strains or experimental RNAi treatments and control animals was calculated, and the statistical significance of that difference was assessed across all three replicates using a two-tailed Student’s t-test.

## Results

### *In silico* analysis of the *C. elegans* PP2A/4/6 phosphoprotein phosphatases

**Fig 1:**
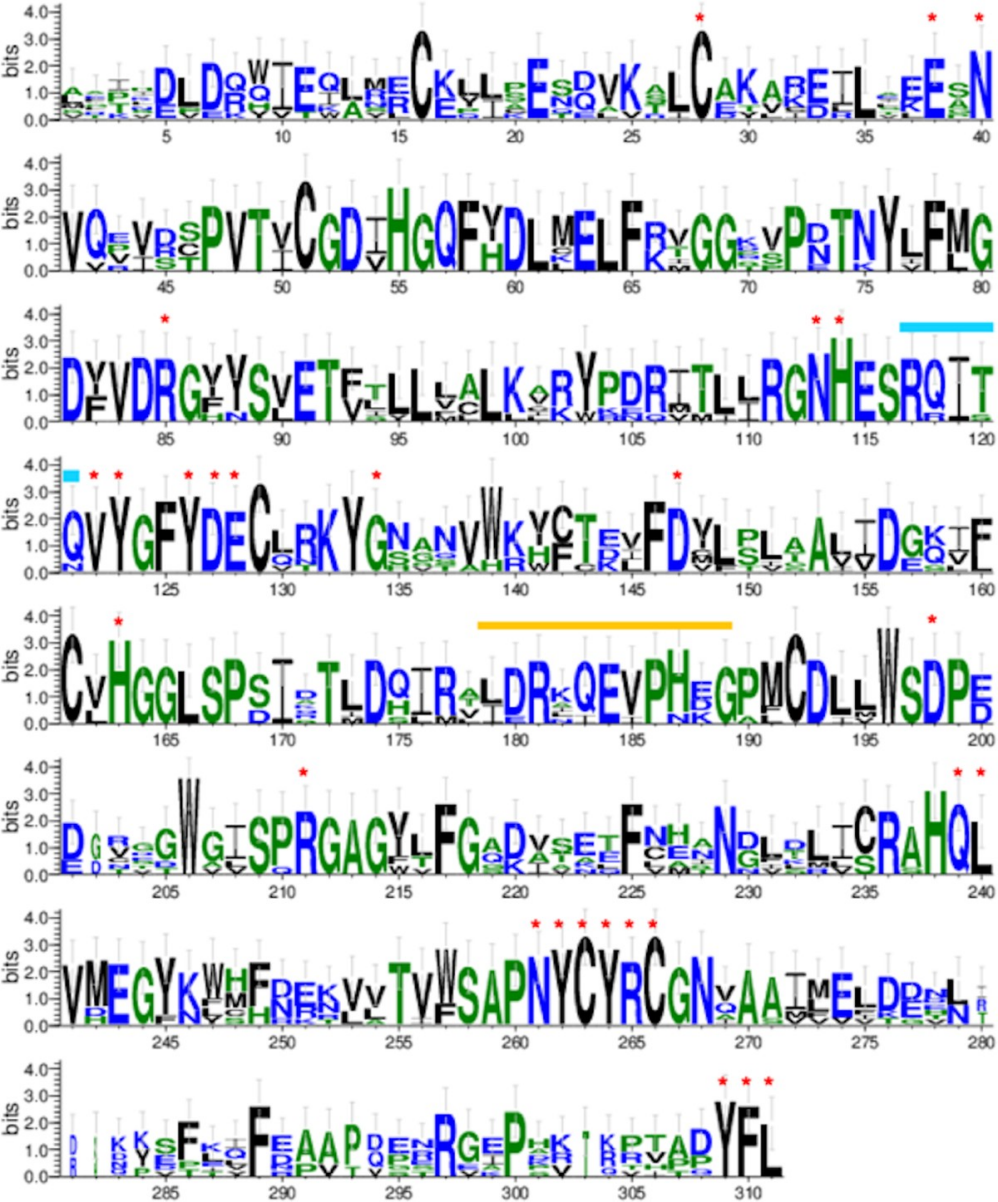
The amino acid sequences of catalytic subunits of the PP2A/4/6 phosphoprotein phosphatase complexes in humans and *C*. *elegans* are highly homologous. The frequency of a particular amino acid at equivalent positions within catalytic subunits of the PP2A, PP4, and PP6 complexes in humans was compared to the corresponding subunits in *C*. *elegans*. When necessary, N-termini were trimmed by as many as 26 amino acids so that all sequences being compared were of the same length. Amino acids are denoted by their single letter code. The height of each letter corresponds to the frequency with which a particular amino acid is found at a given position, with taller letters indicating greater consensus at that position among the proteins being compared. If only one letter appears at a given position it indicates absolute identity at that position between the proteins. Letters are stacked when different amino acids are found at the equivalent position within the proteins. The color of the letters indicates whether amino acids are hydrophilic (blue), neutral (green) or hydrophobic (black). Red asterisks are placed above amino acids with absolute identity shown to be bound or modified by regulatory proteins TIPRL, PTPA, α4 or LCMT-1 in mammals. The conserved helix switch and loop switch regions are indicated by turquoise and orange bars, respectively.

In both yeast and mammals significant structural and functional similarities between members of the PP2A/4/6 family of phosphoprotein phosphatases exist. In addition, proteomic and smaller scale biochemical studies have revealed non-canonical associations between subunits of the holoenzyme complexes [37, 46, 47]. To ask how the PP2A/4/6 proteins in *C*. *elegans* compare to their human counterparts we first took an *in silico* approach. Alignments of the sequences of the three human catalytic subunits PP2Ac, PP4c and PP6c with their *C*. *elegans* orthologs revealed that with the exception of their extreme N-terminal sequences, there is strong homology between them throughout their length (Fig 1; S1 Fig; S1 Table). For example, LET-92 and PPH-4.1 are both more than 80% identical to PP2Ac and PP4c, respectively. Key structural motifs and regulatory residues are especially well-conserved both within catalytic subunits of the PP2A/4/6 family and across species. Notably the YFL tripeptide at the C-terminus that is characteristic of PP2Ac-like phosphatases in many species and that may be a site of regulatory post-translational modifications is conserved in *C*. *elegans*. Both the helix switch and loop switch regions that are required for the binding of the regulatory protein α4 to PP2Ac in mammals also appear to be present within all catalytic subunits of the PP2A/4/6 family in *C*. *elegans*. Based on these similarities, we anticipated that our studies would demonstrate significant parallels between these phosphatases in worms and humans.

We next asked whether *C*. *elegans* PP2A/4/6 proteins were expected to associate with the same binding partners as their human orthologs *in vivo*. To answer this question we performed database searches, using SMK-1 and PPH-4.1 as representative subunits for our analyses. We first used Wormbase to compile a list of PPH-4.1 interactors (S2 Table). Drawing mainly from biochemically verified associations, high throughput yeast two-hybrid data, and interaction networks generated *in silico*, this list captures a broad spectrum of potential interactors. Included among them are not only proteins that could be members of a trimeric complex along with PPH-4.1 but also regulators, functional analogs, and possible substrates that may be dephosphorylated by PPH-4.1. In addition to SMK-1, two other worm homologs of PP4 regulatory subunits, PPFR-1 and PPFR-2, were included as potential PPH-4.1 interactors. The only other worm orthologue of a PP4 regulatory subunit, F46C5.6, was absent from the list of PPH-4.1 interactors found on Wormbase. Both orthologs of the yeast Tip41p-Tap42p regulatory system that modulates all members of the PP2A/4/6 family were identified as PPH-4.1 interactors, namely PPFR-4 and ZK688.9, orthologous to mammalian ⍺4 and TIPRL, respectively. One other notable potential PPH-4.1 interactor is PPH-6, which encodes the *C*. *elegans* orthologue of PP6c, the catalytic subunit of the related but distinct PP6 complex. When we compared the list of potential PPH-4.1 interactors with the list of proteins predicted by Wormbase to interact with SMK-1, we expected to find significant overlap, especially among orthologues of the PP4 complex. Instead, although PPH-4.1 was listed as an interactor of SMK-1 the only other potential PP4 complex member that was in common to the list of interactors for both PPH-4.1 and SMK-1 was the regulatory subunit PPFR-1 (S3 Table).

To gain further insight into the possible relationships between SMK-1, PPH-4.1, and their potential binding partners, we performed interaction network analysis using Genemania. All homologues of the PP2A/4/6 family earmarked as possible interactors of PPH-4.1 or SMK-1 on Wormbase along with the modulator PPFR-4 were included. This analysis yielded additional putative PPH-4.1 interactors (S2 Fig). Among these were PAA-1 and Y71H2AM.20, *C*. *elegans* orthologues of human PR65⍺/PR65β and PTPA, respectively. This was surprising because in human cells these proteins associate with PP2c, the catalytic subunit of the PP2 complex, and not the PP4 complex. Specifically, PR65⍺/PR65β are scaffolding units of the PP2 complex, and PTPA has been shown to modify the catalytic site of the PP2c enzyme [48–50]. This raises the intriguing possibility that certain subunits may associate with both a PP4c-containing complex and with PP2A complexes in *C*. *elegans*, a scenario that is supported by biochemical evidence in mammalian systems [37, 46].

Taken together, our database queries led to the unexpected finding that *C*. *elegans* orthologs of proteins not only of the PP4 complex but also of canonical subunits of the PP2A and PP6 holoenzyme complexes could potentially associate with SMK-1 and PPH-4.1. This lent credence to our experimental strategy of considering all *C*. *elegans* orthologs of the entire PP2A/4/6 family so that evidence supporting putative novel interactions between subunits in the context of aging would not be missed. Our database searches also revealed that evolutionarily conserved proteins that act to regulate the PP2A/4/6 holoenzymes by affecting the assembly of the complexes or by manipulating the active site of the catalytic subunit may also be binding partners of PPH-4.1.

Separate from posttranslational modifications of the PP2A/4/6 family catalytic subunits that influence their affinity for specific regulatory subunits, the catalytic activity of the enzymes is in parallel regulated by the evolutionarily conserved Tip41-Tap42 system. Constituents of this regulatory module may form transient or stable associations with PP2A/4/6 proteins. Originally identified in *Saccharomyces cerevisiae*, both the Tip 41 and Tap 42 proteins function in the TORC pathway [51, 52]. Specifically, Tip41p antagonizes TORC by inhibiting Tap42, a negative regulator of the PP2A-related phosphatase Sit4p that dephosphorylates TORC substrates. In this manner Tip41p functions to activate PP2A-like catalysis by opposing Tap42. The mammalian ortholog of Tip41p, called TIPRL, on the other hand, cooperates with ⍺4 (mammalian Tap42p) to promote mTORC activity by inhibiting the PP2A/4/6 phosphatases [38]. In particular TIPRL and ⍺4 together bind to holoenzymes and displace metal ions from the active site of the catalytic subunit to produce a latent yet stable complex. Catalytic activity of such decommissioned enzymes can be restored by PTPA, which reloads the metal ions back into position [53]. *C*. *elegans* orthologs of all three of these proteins came up in our database searches for PPH-4.1 interactors. Since one of the objectives of our study was to uncover functional evidence that might implicate physical associations between proteins we therefore elected to include them as part of our functional analyses. In total we characterized 20 genes including each *C*. *elegans* ortholog of human PP2A/4/6 subunits along with orthologs of the regulatory proteins TIPRL, ⍺4 and PPTA (Table 1). The names of all human genes encoding catalytic, regulatory, or scaffolding constituents of PP2A/4/6 subfamily complexes as well as genes encoding proteins that regulate the phosphatases through physical associations are listed along with their *C*. *elegans* orthologs in Table 1.

**Table 1:** *C*. *elegans* orthologs of human PP2A/4/6 holoenzyme subunits and regulatory proteins.

		*H*. *sapiens*		*C*. *elegans*	
Complex	Subunits	Gene	Protein	Gene	Protein
**PP2A**	**Catalytic**	*PPP2CA*	Ppp2c⍺/PP2Ac⍺	*let-92*	LET-92
		*PPP2CB*	Ppp2cα/PP2Acα		
	**Scaffolding**	*PPP2R1A*	A⍺/PR65⍺	*paa-1*	PAA-1
		*PPP2R1B*	Aα/PR65α		
	**B/B55 regulatory**	*PPP2R2A*	PR55⍺/B⍺	*sur-6*	SUR-6
		*PPP2R2B*	PR55α/Bα		
		*PPP2R2C*	PR55ɣ/Bɣ		
		*PPP2R2D*	PR55δ/Bδ		
	**B'/B56 regulatory**	*PPP2R5A*	B'⍺/B56⍺/PR61⍺	*pptr-1*	PPTR-1
		*PPP2R5B*	B'α/B56α/PR61α		
		*PPP2R5C*	B'ɣ/B56ɣ/PR61ɣ	*pptr-2*	PPTR-2
		*PPP2R5D*	B'δ/B56δ/PR61δ		
		*PPP2R5E*	B'ε/B56ε/PR61ε	*pptr-1*	PPTR-1
	**B'' regulatory**	*PPP2R3A*	B''⍺/PR72	T22D1.5	T22D1.5
		*PPP2R3B*	B''α/PR70/PR48	F43B10.1	F43B10.1
		*PPP2R3C*	B''ɣ/G5PR	*rsa-1*	RSA-1
	**B‴/striatin regulatory**	*STRN/PPP2R6A*	B‴⍺/STRN	cash-1	CASH-1
		*STRN3/PPP2R6B*	B‴α/STRN/SG2NA		
		*STRN4/PPP2R6C*	B‴ɣ/STRN4	n/a	n/a
**PP4**	**Catalytic**	*PPP4C*	Ppp4c/PP4/PPX	*pph-4*.*1*	PPH-4.1
				*pph-4*.*2*	PPH-4.2
	**Regulatory**	*PPP4R1*	PP4R1	*ppfr-1*	PPFR-1
		*PPP4R2*	PP4R2	*ppfr-2*	PPFR-2
		*PPP4R3⍺*	Ppp4R3⍺	*smk-1*	SMK-1
		*PPP4R3α*	Ppp4R3α	n/a	n/a
		*PPP4R4*	PP4R4	F46C5.6	F46C5.6
**PP6**	**Catalytic**	*PPP6C*	Ppp6c/PP6	*pph-6*	PPH-6
	**Regulatory**	*PPP6R1*	Ppp6R1/PP6R1	*saps-1*	SAPS-1
		*PPP6R2*	Ppp6R2/PP6R2		
		*PPP6R3*	Ppp6R3/PP6R3		
		*ANKRD28*	PP6-ARS-A/PPP1R65	n/a	n/a
		*ANKRD44*	PP6-ARS-B		
		*ANKRD52*	PP6-ARS-C		
**PP2A/4/6 activating or inactivating proteins**		⍺4/IGBP	⍺4/IGBP	*ppfr-4*	PPFR-4
		*TIPRL*	TIPRL	ZK688.9	ZK688.9
		*PPP2R4/PTPA*	PTPA/PR53	Y71H2AM.20	Y71H2AM.20

### Functional characterization of SMK-1 during aging

To investigate the PP2A/4/6 family of protein phosphatases during aging in *C*. *elegans* we began by studying the PP4 complex. We chose to focus on the SMK-1 regulatory subunit in particular as a test case to validate our experimental approach based on reverse genetics because direct binding between SMK-1 and the catalytic subunit PPH-4.1 had been demonstrated *in vitro* with recombinant proteins [14].

In light of this connection, we expected that if SMK-1 functions as part of a PP4 complex in adult animals, RNAi knockdown of *smk-1*, *pph-4*.*1*, and potentially other constituent subunits of the complex should all result in similar if not identical phenotypes. Moreover, if a SMK-1/PPH-4.1-containing PP4 complex regulates DAF-16 during aging then RNAi targeting subunits of that complex should phenocopy RNAi targeting *daf-16*. Our strategy was to first compile a set of phenotypes attributable to *smk-1* inhibition in adult animals. These phenotypes would then generate a metric to which other genes encoding *C*. *elegans* orthologs of human PP4 complex members could be compared to determine the likelihood of them cooperating to produce the same output, including regulating the age-dependent increase in transcriptional activity of DAF-16. Further, functional evidence indicating that particular subunits act together would imply that they associate with each other *in vivo*. We anticipated that the same comparative analysis could be applied to putative constituents of the PP2A and PP6 complexes to determine whether they might also function during aging in *C*. *elegans*. Because we were interested in characterizing the SMK-1/PPH-4.1-containing complex in the context of aging, we conducted all of our studies on adult worms at Day 6 of adulthood when DAF-16 is transcriptionally active in parallel to control L4 larval stage animals [54]. A two-tiered panel of assays was applied to evaluate the function of SMK-1 during aging. First, we asked whether SMK-1 is necessary for the transcriptional activity of DAF-16 in adult *C*. *elegans* by examining the expression of an *in vivo* reporter of DAF-16 activity, *Plys-7*::*GFP* in animals treated with RNAi targeting *smk-1*. We then functionally characterized *smk-1* by assessing its contribution to the ability of animals to resist acute environmental insults including bacterial infection with *Pseudomonas aeruginosa* (PA14), exposure to elevated temperature, and irradiation with ultraviolet (UV) light. In each stress assay, the phenotype resulting from *smk-1* knockdown was compared to the effect of knocking down *daf-16*.

**Fig 2:**
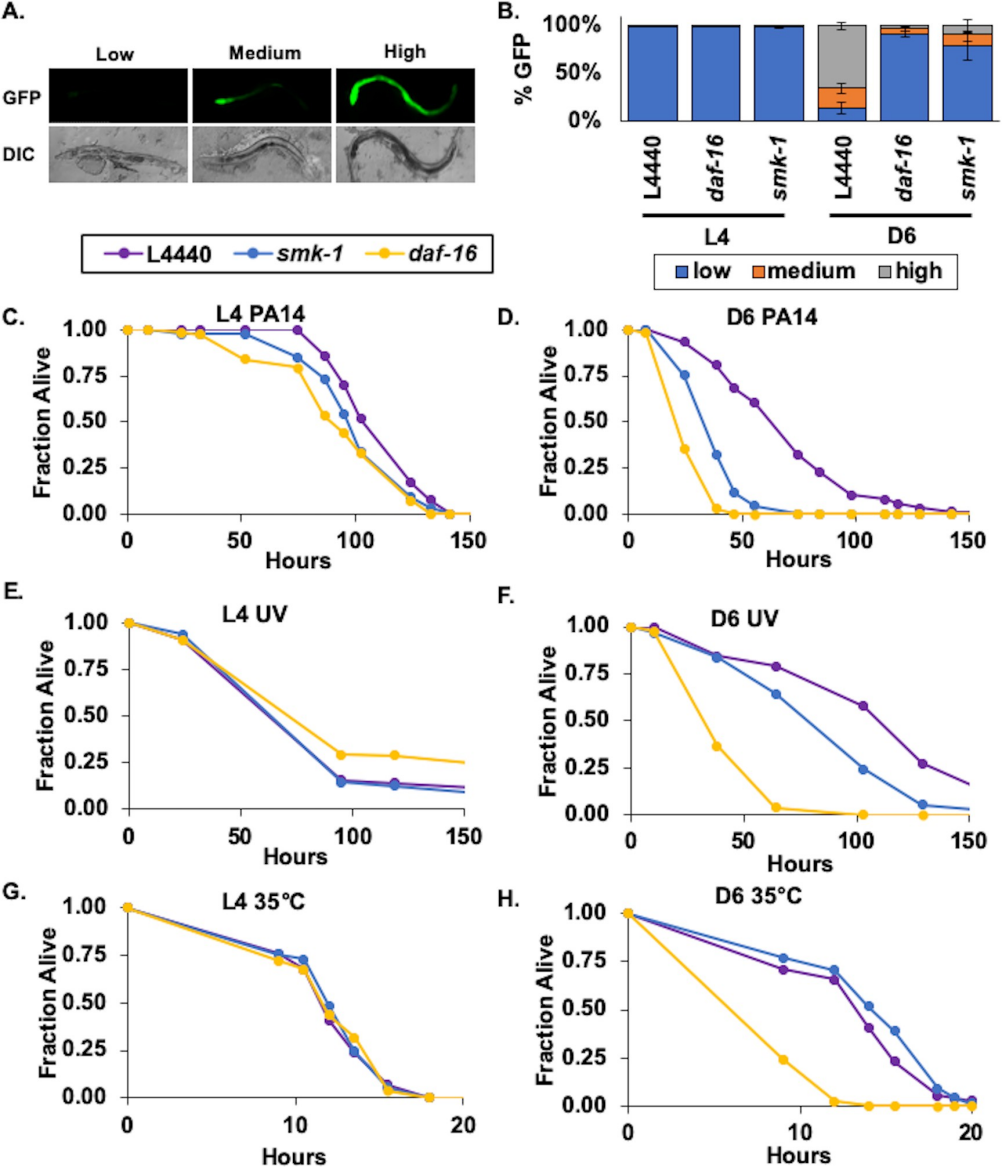
SMK-1 confers resistance to bacterial infection and ultraviolet radiation in adult *C*. *elegans*. (A) Animals expressing the *Plys7*::*gfp in vivo* reporter for DAF-16 transcriptional activity were examined by fluorescence microscopy at D6 of adulthood. Age-synchronized isogenic animals could be assigned to one of three categories based on the relative expression of GFP observed in the intestine. “Low”: little to no GFP expression “Medium”: GFP expression most apparent in the anterior portion of the intestine but also at lower levels toward the midbody. “High”: robust GFP expression along the entire length of the intestine. DIC, differential interference contrast image corresponding to the fluorescence image (GFP) shown above. (B) Quantification of worms in the Low (blue), Medium (orange), and High (grey) categories of *Plys7*::*gfp* expression at the L4 stage and D6 of adulthood following RNAi treatment targeting either *daf-16* or *smk-1*. n>200 animals for each RNAi treatment in three biological replicates. The average number of worms in each category is shown with error bars representing standard deviation from the mean. (C and D) Results of *Pseudomonas aeruginosa* infection assays. The survival of *C*. *elegans* subjected to RNAi against indicated genes beginning at L1 and exposed to *P*. *aeruginosa* continuously beginning at L4 (C) or D6 of adulthood (D) is plotted as the fraction of animals alive as a function of time. (E and F) Results of UV irradiation assays. Survival curves for *C*. *elegans* subjected to RNAi against the indicated genes from L1 until death and exposed to 1000 J/m^2^ UV radiation at L4 (E) or D6 of adulthood (F). Hours represent time elapsed since the end of the irradiation treatment. (G and H) Results of heat stress assays. Survival curves for RNAi-treated *C*. *elegans* incubated at 35°C beginning at L4 (G) or D6 of adulthood (H) until they died. Hours represent the total time worms were incubated at 35˚ C. Beginning at the L1 stage, worms were maintained on RNAi plates without interruption for the duration of their lives. n>90 animals for each RNAi treatment in three biological replicates. Purple, L4440; yellow, *daf-16* RNAi; blue, yellow, *smk-1* RNAi.

We expected that if SMK-1 is necessary for the function of DAF-16 during adulthood, just as it is in the *daf-2(e1370)* mutant background, then RNAi targeting either *smk-1* or *daf-16* would have similar consequences. With minor exceptions, this is what we found. After initiating RNAi to knockdown *smk-1* or *daf-16* at the L1 larval stage, we first monitored the expression of the *Plys7*::*gfp* reporter over time by fluorescence microscopy (Fig 2A). Compared to worms at the L4 larval stage, the expression of GFP driven by the promoter of *lys-7* in the intestines was substantially higher in Day 6 adults, consistent with an age-dependent increase in the transcriptional activity of DAF-16. In examining a population of animals we were able to assign individuals to one of three categories based on their GFP expression pattern (Fig 2A). In general, we observed robust and uniform GFP expression along the length of the intestine among the majority of individuals in a synchronized population of Day 6 adults. These animals were scored as “high” GFP expressers. However, in a proportion of animals high levels of GFP expression were primarily confined to a few anterior intestinal cells with progressively weaker GFP expression in more posterior cells. Animals with this pattern of GFP expression were classified as “medium” GFP expressers. An even smaller fraction of worms expressed low levels of intestinal GFP that was frequently difficult to detect. RNAi targeting *daf-16* or *smk-1* had no effect on expression levels of the *Plys7*::*gfp* reporter in L4 larvae. By Day 6 of adulthood, however, *daf-16* knockdown resulted in a significant decrease in GFP expression (Fig 2B). This suggests that DAF-16 becomes activated in an age-dependent manner, consistent with previous studies [8–10]. Recapitulating our prior observations, knocking down *smk-1* also resulted in decreased reporter expression in Day 6 adults (Fig 2B), and so together our results indicate that the transcriptional activity of DAF-16 during aging in non-stressed wildtype animals may be regulated by SMK-1.

Continuing to examine the parallels between the function of SMK-1 under low IIS conditions and during normal aging, we next asked whether SMK-1 is required to confer resistance to environmental stress in adult *C*. *elegans*. SMK-1 expression was inhibited by RNAi, and the ensuing effect on the ability of L4 larvae and Day 6 adult *C*. *elegans* to resist environmental stress was assessed by measuring the survival of animals challenged with an acute insult. While RNAi targeting *smk-1* mildly enhanced the susceptibility of L4 larvae to *P*. *aeruginosa* infection, it had a more substantial impact on pathogen susceptibility during adulthood, reducing the median lifespan of infected Day 6 adult animals to almost half that of untreated controls (Fig 2C and 2D). This was comparable to the effect of knocking down *daf-16* on the resistance of adult animals and corroborates our previous results (Fig 2D and [10]). The age-dependency of phenotypes resulting from *smk-1* knockdown became even more apparent when RNAi-treated worms were subjected to UV radiation. Similar to when *daf-16* was targeted by RNAi, *smk-1* knockdown shortened the survival of *C*. *elegans* irradiated at Day 6 of adulthood, but it was inconsequential for animals irradiated as L4 larvae (Fig 2E and 2F). Examining the thermotolerance of RNAi-treated worms by warming them to 35˚C revealed that unlike *daf-16*, *smk-1* is not important for thermotolerance in either L4 larvae or Day 6 adults, consistent with the role of SMK-1 in *daf-2(e1370)* mutants (Fig 2G and 2H and [12, 13]). The results of our stress assays demonstrate that like DAF-16, SMK-1 contributes to stress resistance in an age-dependent manner and that it is important for the innate immunity and protection from UV radiation in adult *C*. *elegans*. The phenotypic similarities resulting from the knockdown of either gene suggests that their products may function within the same pathway.

### Putative subunits of the PP4 and PP2A complexes influence the transcriptional activity of DAF-16 in adult *C. elegans*

**Fig 3:**
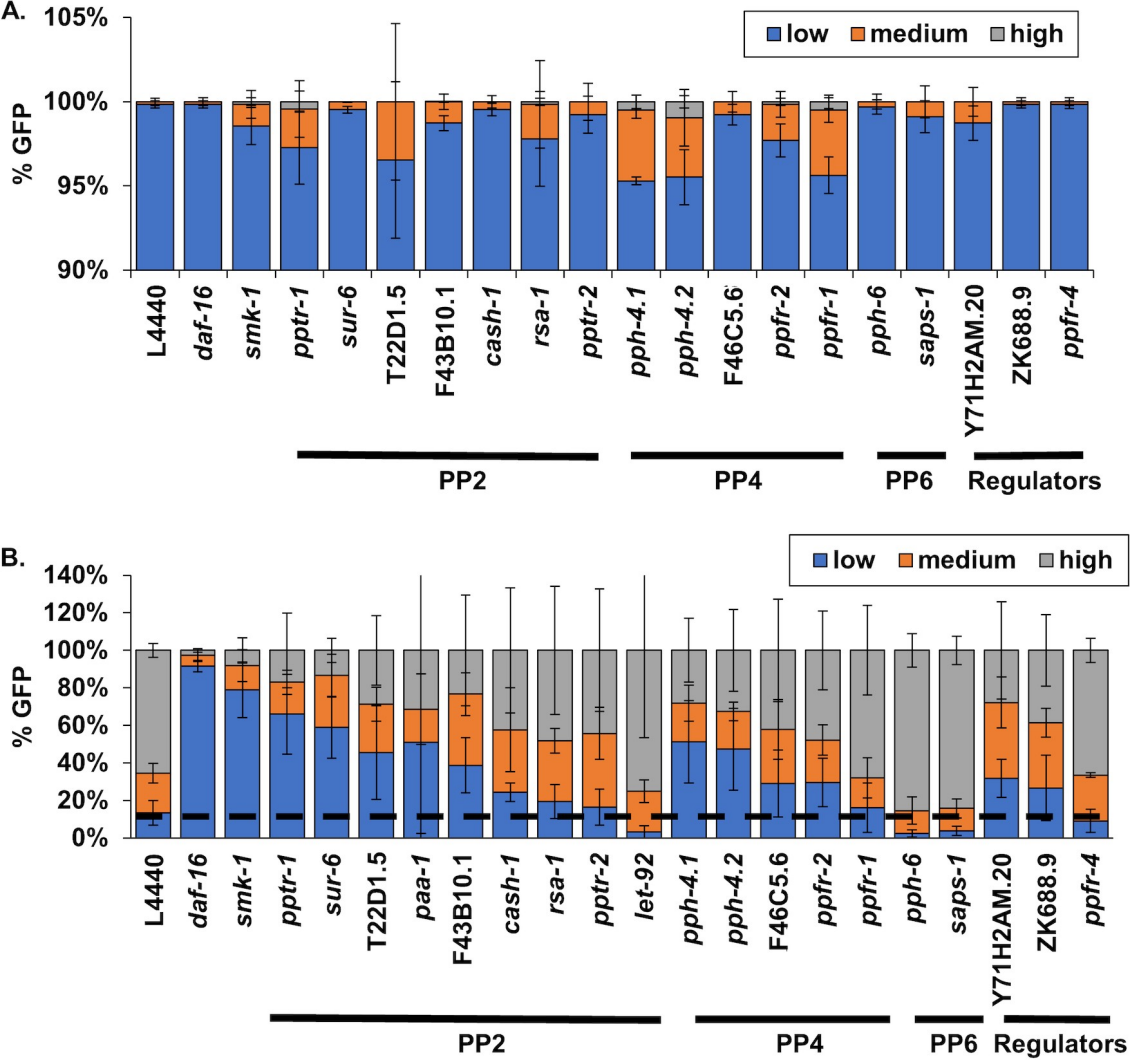
Genes encoding putative members of the PP2A, PP4, and PP6 complexes are required for the age-dependent increase in DAF-16 transcriptional activity. Quantification of worms in the Low (blue), Medium (orange), and High (grey) categories of *Plys7*::*gfp* expression at the L4 stage (A) and D6 of adulthood (B) following RNAi treatment targeting the indicated gene. n>200 animals for each RNAi treatment in three biological replicates. The average number of worms in each category is shown with error bars representing standard deviation from the mean.

To expand our analyses to include all putative subunits of the PP4 complex and, in fact, to the entire PP2A/4/6 family, we first surveyed the effect of knocking down each gene individually on the age-dependent increase in *Plys7*::*gfp* expression (Fig 3). RNAi treatments that resulted in greater than 25% of worms being assigned to the low GFP expression category were considered to function in a similar manner to SMK-1 to regulate the activity of DAF-16. None of the genes that we tested had a significant effect on the expression of the *Plys7*::*gfp* reporter in L4 larvae (Fig 3A). At Day 6, knockdown of the PP4 catalytic subunit *pph-4*.*1* and its paralog *pph-4*.*2* had some of the strongest effects on GFP expression, resulting in a higher proportion of worms in the low expressing category, similar to the effect of knocking down *smk-1* (Fig 3B). This result was expected since SMK-1 and PPH-4.1/PPH-4.2 associate *in vivo* [13, 14]. RNAi targeting F46C5.6 and *ppfr-2*, *C*. *elegans* homologues of two other PP4 regulatory subunits, also modestly increased the proportion of worms expressing low levels of GFP, yet this effect was not statistically significant. Notably, inhibiting the expression of several worm homologues of PP2 complex members also suppressed the age-dependent increase in *plys7*::*gfp* expression. Inhibition of *pptr-1*, *sur-6*, T22D1.5, *paa-1*, and F43B10.1 all resulted in an increase in low levels of GFP expression at Day 6 relative to control animals. It bears mentioning that in our initial attempts to knock down the PP2A complex orthologs *let-92* and *paa-1* starting at the L1 larval stage we observed developmental phenotypes, and so for the *plys-7*::*gfp* expression assay and for the functional assays described below, we allowed animals to reach the L4 larval stage before treating them with RNAi targeting *let-92* or *paa-1*. In addition to genes encoding orthologs of PP4 and PP2A complex subunits, one other gene that was found to be necessary for the age-dependent increase in *plys-7*::*GFP* is Y71H2AM.20, the *C*. *elegans* ortholog of the regulatory protein PTPA. These results suggest that SMK-1 and PPH-4.1 function together in adult *C*. *elegans*, and they imply roles for both the PP4 and PP2A complexes in regulating the transcriptional activity of DAF-16 during aging.

### Members of the PP2A/4/6 subfamily contribute to innate immunity during aging

**Fig 4:**
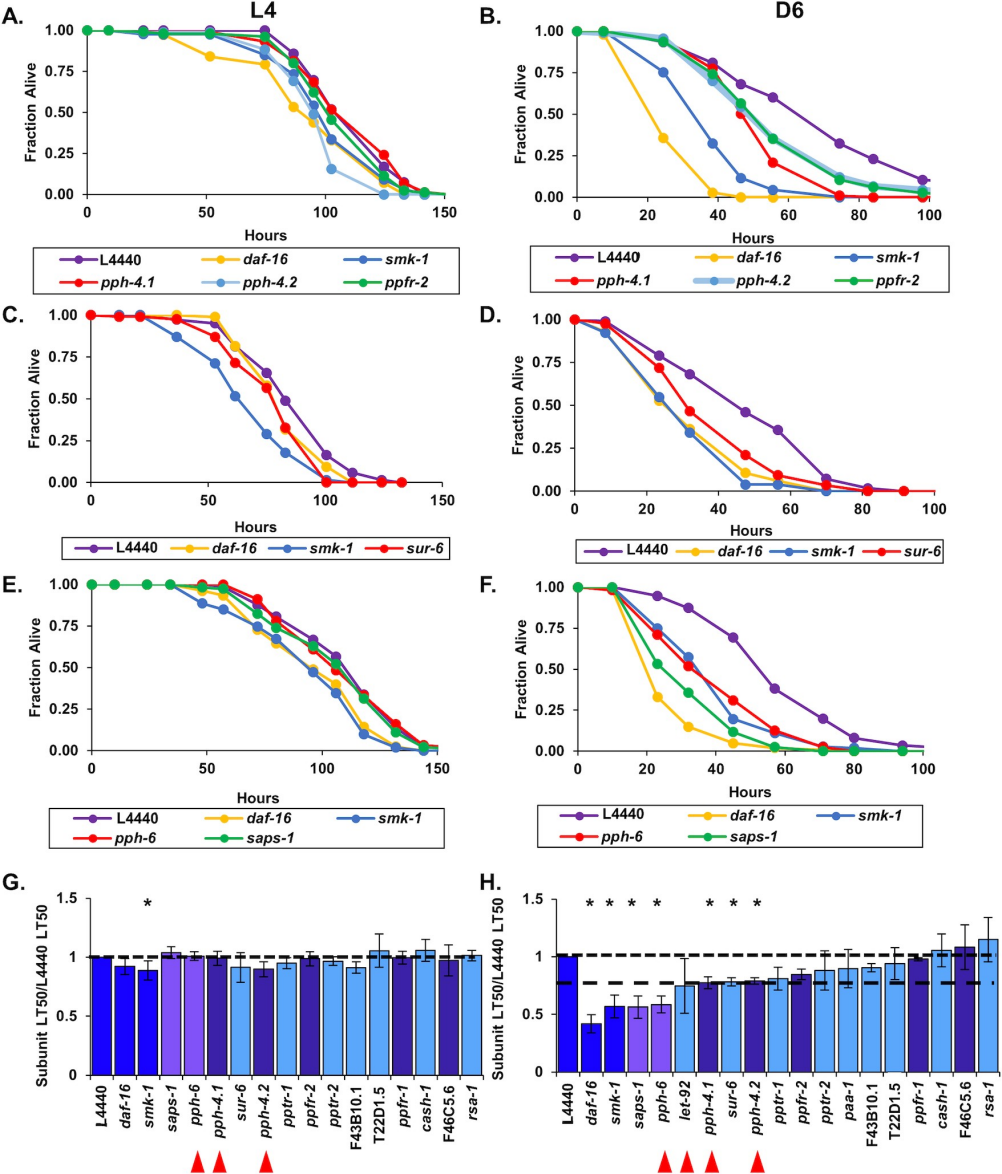
Members of putative PP4 and PP6 holoenzymes along with the PP2A regulatory subunit SUR-6 are required for innate immunity during adulthood in *C*. *elegans*. Following RNAi treatment beginning at the L1 stage to target *C*. *elegans* homologs of catalytic and regulatory subunits of the PP4 (A and B), PP2A (C and D), and PP6 (E and F) complexes, worms were infected with *P*. *aeruginosa* at the L4 larval stage (A,C,E) or at D6 of adulthood (B,D,F). The fraction of worms alive at each time point after infection was initiated is plotted as a function of time in hours. In all cases RNAi targeting *daf-16* or *smk-1* and the empty RNAi vector L4440 were included as controls. Only RNAi knockdowns that produced statistically significant phenotypes are shown. (G and H) The average relative median survival (LT_50_) of animals treated with RNAi targeting the indicated genes following infection with *P*. *aeruginosa* at L4 (G) or D6 (H) is shown as a fraction of the average median survival of L4440 controls. Bars, standard error of the mean (SEM). Bar colors correspond to the protein phosphatase complex to which products of the indicated genes belong or to controls. Dark blue: L4440, *daf-16*, and *smk-1*; light blue: PP2A; dark purple: PP4; light purple: PP6. Asterisks indicate RNAi treatments producing statistically significant differences in median survival (p<0.05). Horizontal lines are drawn at a relative median survival of 1 and, for a reference in (H) the relative median survival of adult worms treated with RNAi against *pph-4*.*1*. Red arrowheads are beneath the names of genes encoding catalytic subunits of the PP2A, 4 and 6 complexes.

We found that *smk-1* is necessary to confer resistance to bacterial infection in Day 6 adults, as is DAF-16 (Fig 2D). This result leads to the prediction that *pph-4*.*1*, encoding the catalytic subunit of the PP4 complex, is also required for host defense during aging. Further, based on the results of our *in vivo* reporter assay for DAF-16 transcriptional activity, we wondered whether PP2A subunits may also function in innate immunity later in life. We addressed these possibilities as part of our characterization of the PP2A/4/6 family in adults challenged with *P*. *aeruginosa*. In a phenotype reminiscent of the *smk-1* knockdown, RNAi targeting homologues of the PP4 catalytic subunit *pph-4*.*1* or its paralog *pph-4*.*2* reduced the ability of Day 6 adults to resist bacterial infection but had no effect on the susceptibility of L4 larvae to pathogen (Fig 4A, 4B, 4G and 4H). The magnitude of the resulting decrease in median lifespan (LT_50_) of infected animals caused by inhibiting *pph-4*.*2* expression was comparable to the effect of *smk-1* knockdown, but *RNAi* targeting *pph-4*.*1* had a considerably milder effect across our replicates (Table 2). Coupled with the results of our *in vivo* reporter assay, this result represents a second independent circumstance in which RNAi inhibition of *smk-1* phenocopied the knockdown of *pph-4*.*1/4*.*2*. We also considered it as providing validation to our experimental approach since our functional analyses confirmed biochemically verified interactions between SMK-1 and PPH-4.1/4.2.

**Table 2:** Summary of phenotypes resulting from knockdown of members of the PP2A/4/6 family and their regulators in wildtype N2 *C*. *elegans*.

		*P*. *aeruginosa* Infection				Heat Stress				UV Irradiation			
		L4		Day 6		L4		Day 6		L4		Day 6	
		Average relative LT50	p-Value	Average relative LT50	p-Value	Average relative LT50	p-Value	Average relative LT50	p-Value	Average relative LT50	p-Value	Average relative LT50	p-Value
**PP2A**	***let-92***	NA	NA	0.746	2.7E-01	NA	NA	0.866	1.8E-01	NA	NA	0.605	**1.4E-02**
	paa-1*	NA	NA	0.897	4.7E-01	NA	NA	0.802	1.5E-01	NA	NA	0.767	**2.5E-02**
	pptr-1*	0.949	2.7E-01	0.809	1.1E-01	0.940	2.5E-01	0.866	1.9E-01	0.909	7.5E-01	0.620	**9.1E-03**
	sur-6*	0.913	4.3E-01	0.784	**1.3E-02**	0.822	3.5E-01	0.861	**7.4E-03**	1.192	3.2E-01	0.836	3.8E-01
	***cash-1***	1.057	4.7E-01	1.054	6.4E-01	1.131	6.2E-01	1.015	6.5E-01	0.846	2.9E-01	0.858	3.4E-01
	***pptr-2***	0.966	3.3E-01	0.881	4.2E-01	0.949	5.9E-01	0.921	3.5E-01	0.984	9.2E-01	0.958	7.4E-01
	***rsa-1***	1.014	7.1E-01	1.150	3.9E-01	0.937	6.1E-01	1.111	2.4E-01	2.664	4.5E-01	1.005	7.4E-01
	F43B10.1*	0.912	1.2E-01	0.906	6.5E-02	0.979	8.9E-01	1.022	6.4E-01	0.933	6.5E-01	0.977	6.9E-01
	T22D1.5*	1.055	6.3E-01	0.941	6.1E-01	1.053	6.9E-01	1.075	4.4E-01	0.911	5.4E-01	1.090	3.8E-01
**PP4**	*pph-4*.1*	0.991	8.5E-01	0.922	**2.5E-02**	0.850	**1.7E-03**	1.057	4.6E-01	0.959	8.8E-01	0.915	5.5E-01
	*pph-4*.2*	0.898	1.5E-01	0.526	**8.6E-03**	0.922	3.7E-01	0.954	4.4E-01	0.756	3.7E-01	0.869	2.9E-01
	smk-1*	0.887	**4.7E-04**	0.570	**1.7E-10**	0.985	6.3E-01	0.904	1.3E-01	0.878	8.2E-02	0.707	**9.8E-04**
	***ppfr-2***	0.987	7.9E-01	0.886	**4.0E-02**	1.252	4.2E-01	1.200	2.3E-01	1.457	3.1E-01	1.016	9.0E-01
	***ppfr-1***	0.995	9.1E-01	0.950	1.3E-01	1.017	1.3E-01	0.788	4.6E-01	1.753	4.1E-01	1.037	6.5E-01
	**F46C5.6**	0.972	7.9E-01	1.083	6.0E-01	1.270	4.2E-01	1.269	**3.3E-02**	0.925	6.0E-01	1.164	4.3E-01
**PP6**	***pph-6***	1.009	7.6E-01	0.585	**1.5E-02**	1.100	**1.8E-02**	0.725	1.6E-01	0.806	3.4E-01	0.780	8.9E-02
	***saps-1***	1.037	4.1E-01	0.563	**2.4E-02**	1.024	8.5E-01	0.948	2.1E-01	0.917	6.1E-01	0.855	3.0E-01
**Regulators**	Y71H2AM.20*	1.060	2.3E-01	0.771	1.1E-01	1.376	1.8E-01	0.905	1.6E-01	1.002	1.0E+00	0.831	2.0E-01
	***ppfr-4***	1.098	**3.8E-02**	0.843	2.4E-01	0.989	4.5E-01	0.929	2.4E-01	2.106	3.8E-01	0.674	5.5E-02
	**ZK688.9**	0.990	8.9E-01	0.909	4.9E-01	1.218	3.6E-01	1.115	1.9E-01	0.926	7.3E-01	1.152	1.1E-01

* denotes genes that are required for the increase in *Plys-7*::*GFP* expression at Day 6. Average relative LT_50_ was calculated by dividing the average LT_50_ of RNAi-treated animals in a given stress assay by the average LT_50_ of age-matched control animals in that same assay and thus represents the proportional increase or decrease in median lifespan under stressful conditions that are attributable to RNAi inhibition of the gene of interest. The average relative LT_50_ values corresponding to knockdowns that decreased or increased median lifespan after exposure to stress by more than 10% compared to empty vector control are shaded in orange and blue, respectively. Statistical significance was determined by Student’s t-test, with a threshold of p<0.05. p-values associated with significant susceptibility and resistance are bolded.

Along with *pph-4*.*1/4*.*2* and *smk-1*, RNAi inhibition of another PP4 regulatory subunit homologue, *ppfr-2* (PP4R2 in humans), also reduced the median lifespan of worms infected at Day 6 of adulthood but had no effect on the ability of L4 larvae to resist infection (Fig 4A, 4B, 4G and 4H). No other PP4 regulatory subunits were found to be required for innate immunity in Day 6 adults (Fig 4H; S3 Fig). These results imply that PPFR-2 may act in conjunction with SMK-1 and PPH-4.1 to protect adult *C*. *elegans* from bacterial pathogens.

We found genes encoding putative subunits of other protein phosphatase complexes to also be important for host defense in adult *C*. *elegans*. In addition to the effect of inhibiting the expression of PP4 subunits, one of the ten RNAi treatments directed against *C*. *elegans* orthologs of PP2A subunits enhanced the susceptibility of adult animals to *P*. *aeruginosa* infection. Knockdown of *sur-6*, the sole worm homolog of human PP2A B/B55 regulatory subunits, significantly reduced the median lifespan of infected Day 6 adults but had no effect on larvae (Fig 4C, 4D, 4G and 4H; Table 2). We found no evidence of a role for other orthologs of PP2A complex subunits in adult or larval innate immunity (Fig 4H; S4 Fig). In an unexpected result, inhibiting the expression of two orthologues of PP6 complex members *pph-6*, the catalytic subunit, and *saps-1*, a regulatory subunit, accelerated the rate of death from infection when worms were challenged with *P*. *aeruginosa* at Day 6 of adulthood but not at the L4 larval stage (Fig 4E, 4F, 4G and 4H). This was surprising because none of the PP6 members appear to be required for the increased expression of *plys7*::*gfp* in adults, a departure from *smk-1*-like phenotypes (Fig 3B). Taken together, our results indicate that the entire PP2A/4/6 family plays a role in innate immunity during aging and that the PP6 complex may do so without influencing the transcriptional output of DAF-16.

### SMK-1 and the PP2A complex confer UV resistance in postreproductive *C. elegans*

**Fig 5:**
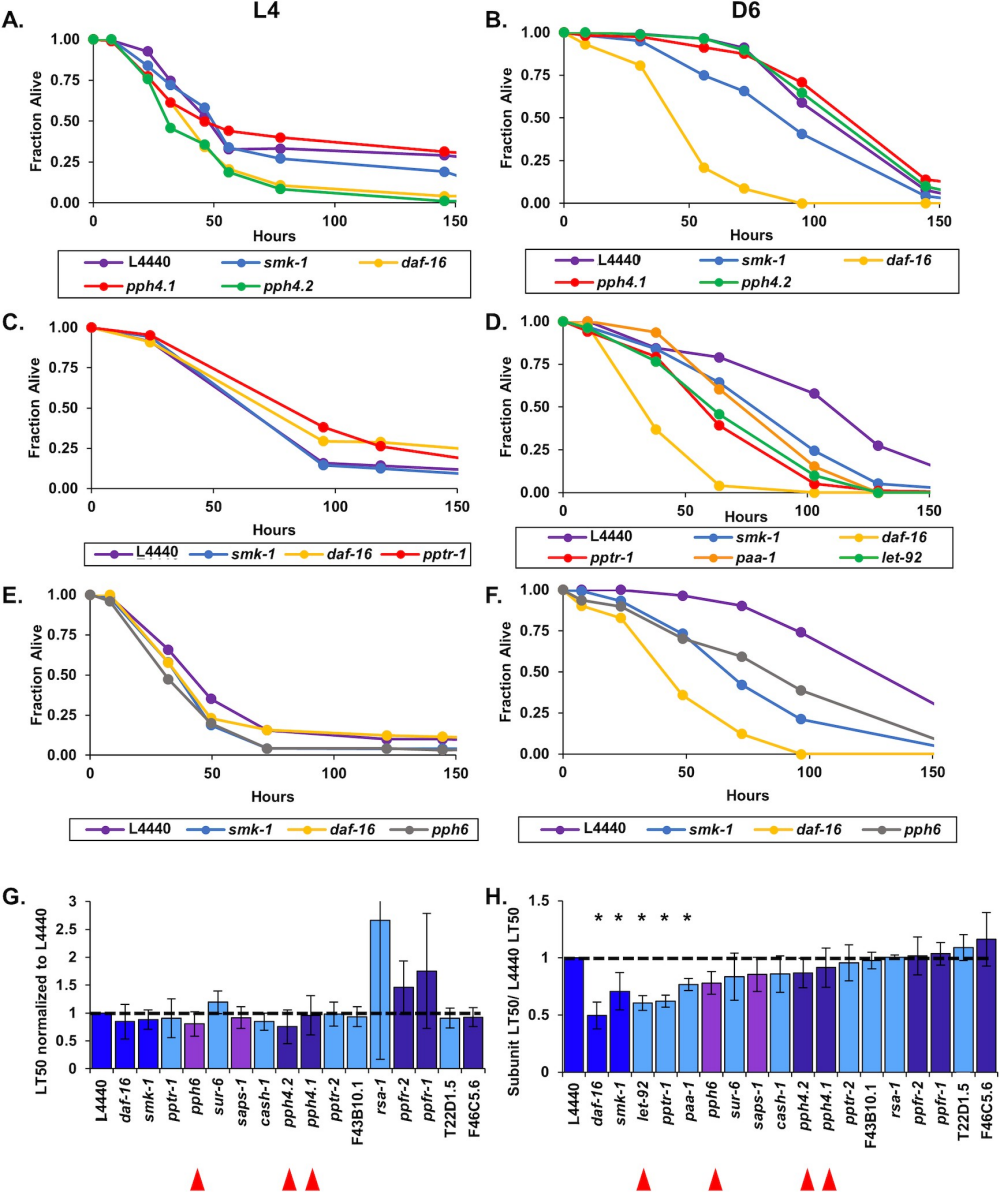
Resistance to UV irradiation in adult *C*. *elegans* is conferred by the PP2A complex and SMK-1. RNAi treatment was initiated at the L1 stage to target *C*. *elegans* homologs of catalytic and regulatory subunits of the PP4 (A and B), PP2A (C and D), and PP6 (E and F) complexes and was continued for the duration of the assay. Worms were exposed to UV radiation at the L4 larval stage (A,C,E) or at D6 of adulthood (B,D,F) after which their survival under standard culturing conditions was monitored. The fraction of worms alive at each time point following the UV treatment is plotted as a function of time. In all cases RNAi targeting *daf-16* or *smk-1* and the empty RNAi vector L4440 were included as controls. Only RNAi knockdowns that produced statistically significant phenotypes are shown. (G and H) The average relative median survival (LT_50_) of animals treated with RNAi targeting the indicated genes following exposure to UV light at L4 (G) or D6 (H) is shown as a fraction of the average median survival of L4440 controls. Bars, standard error of the mean (SEM). Bar colors correspond to the protein phosphatase complex to which products of the indicated genes belong or to controls. Dark blue: L4440, *daf-16*, and *smk-1*; light blue: PP2A; dark purple: PP4; light purple: PP6. Asterisks indicate RNAi treatments producing statistically significant differences in median survival (p<0.05). Horizontal lines are drawn at a relative median survival of 1. Red arrowheads are beneath the names of genes encoding catalytic subunits of the PP2A, 4 and 6 complexes.

We found SMK-1 to be important not only for host defense in Day 6 animals, but also for resistance to ultraviolet irradiation, consistent with its role in *daf-2(e1370)* mutants (Fig 2F; Table 2) [12, 55]. We therefore expected that other components of the PP4 complex would be required for survival upon exposure to UV light during aging. This was not the case. No canonical members of the PP4 holoenzyme, including the catalytic subunits, seem to contribute to UV tolerance in *C*. *elegans*, as knocking them down did not affect the survival of irradiated L4 larvae or Day 6 adults (Fig 5A, 5B, 5G and 5H; S5 Fig). On the other hand, inhibiting expression of the PP2A catalytic subunit, *let-92*, the scaffolding protein *paa-1* and the regulatory subunit *pptr-1* caused Day 6 adult animals exposed to UV light to die more rapidly, leading to significant reductions in their median lifespans (Fig 5C, 5D, 5G and 5H; Table 2). Other orthologs of PP2A subunits do not appear to play a role in conferring resistance to UV light (S6 Fig). Targeting PP6 complex subunits by RNAi was also inconsequential to the ability of adults to resist UV irradiation (Fig 5E, 5F, 5G and 5H; S7 Fig). Our data therefore suggest that a complete PP2A holoenzyme is involved in the ability of post-reproductive animals to resist UV exposure and that it may act in conjunction with SMK-1.

### The PP2A regulatory subunit SUR-6 promotes resistance to heat stress in adult worms

**Fig 6:**
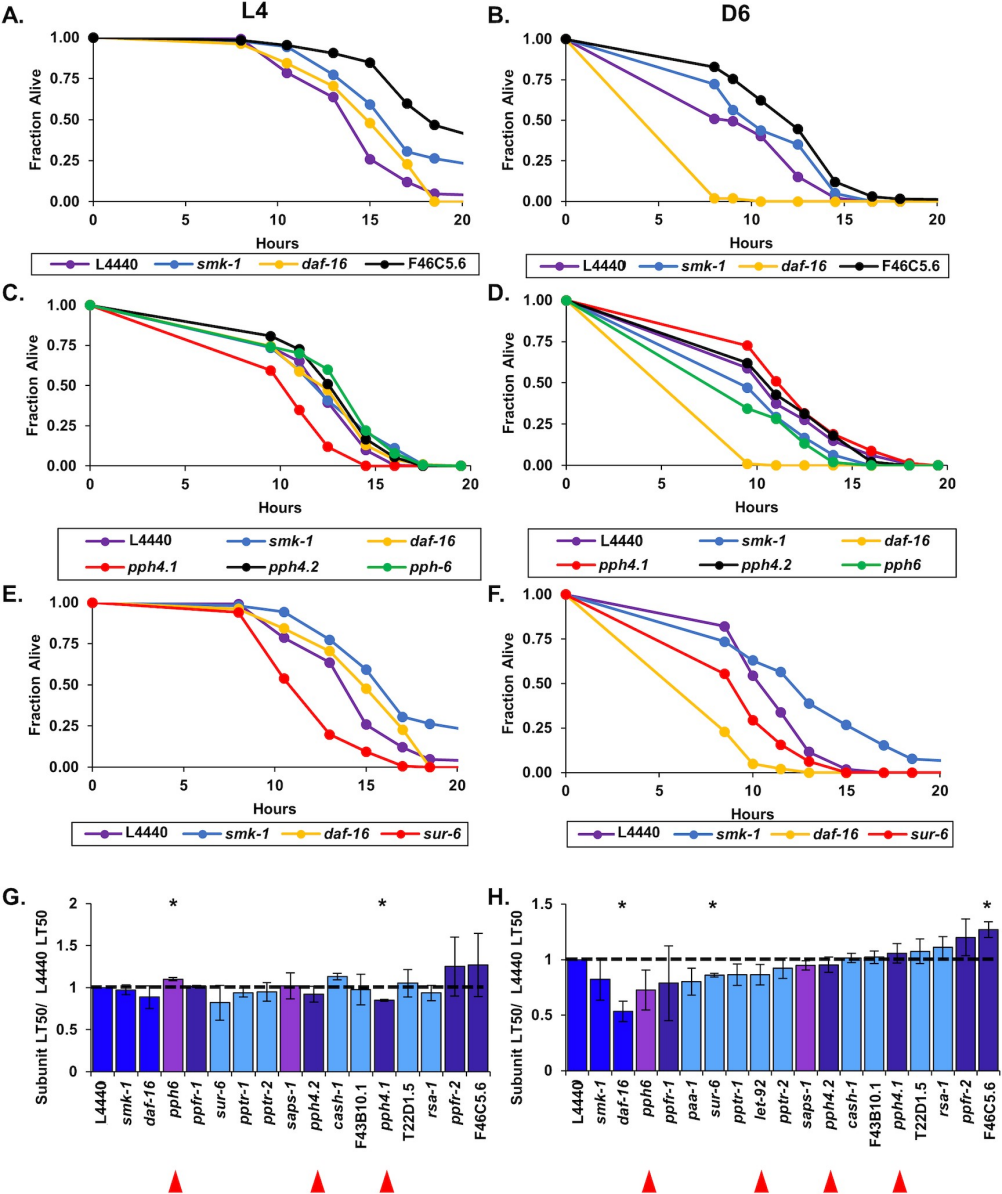
The PP2A subunit SUR-6 contributes to thermotolerance in adult worms. RNAi treatment targeting *C*. *elegans* homologs of catalytic and regulatory subunits of the PP4 (A-D), PP6 (C and D), and PP2A (E and F) complexes was initiated at the L1 stage and continued for the duration of the assay. To induce thermal stress at the L4 larval stage (A,C,E) or at D6 of adulthood (B,D,F) worms were shifted from 20˚ C to 35˚ C until all of the animals had died. The fraction of worms alive at each time point during the incubation at high temperature is plotted as a function of time in hours. In all cases RNAi targeting *daf-16* or *smk-1* and the empty RNAi vector L4440 were included as controls. Only RNAi knockdowns that produced statistically significant phenotypes are shown. (G and H) The average relative median survival (LT_50_) of animals treated with RNAi targeting the indicated genes following the shift to 35˚ C at L4 (G) or D6 (H) is shown as a fraction of the average median survival of L4440 controls. Bars, standard error of the mean (SEM). Bar colors correspond to the protein phosphatase complex to which products of the indicated genes belong or to controls. Dark blue: L4440, *daf-16*, and *smk-1*; light blue: PP2A; dark purple: PP4; light purple: PP6. Asterisks indicate RNAi treatments producing statistically significant differences in median survival (p<0.05). Horizontal lines are drawn at a relative median survival of 1. Red arrowheads are beneath the names of genes encoding catalytic subunits of the PP2A, 4 and 6 complexes.

Despite its roles in conferring resistance to bacterial pathogens and ultraviolet irradiation, SMK-1 does not contribute to thermotolerance in adult *C*. *elegans* even though DAF-16 does (Fig 2G and 2H). Neither of the other two *C*. *elegans* orthologs of PP4 regulatory subunits PPFR-1 and PPFR-2 appear to influence survival of heat stressed animals at the larval or adult stages (Fig 6G and 6H; S8 Fig). In like manner, we found no evidence to suggest that the PP4 catalytic subunits PPH-4.1 or PPH-4.2 function to protect animals from thermal stress during aging, although PPH-4.1 confers thermal tolerance to L4 larvae (Fig 6C, 6D, 6G and 6H). RNAi targeting a putative regulatory subunit F46C5.6, the ortholog of PP4R4, resulted in a surprising modest but significant increase in resistance to high temperatures in Day 6 adult worms but not larvae (Fig 6A, 6B, 6G and 6H). On its own this result suggests that in contrast to the roles of other PP4 regulatory subunits, F46C5.6 could function to inhibit the catalytic activity of the PP4 complex in adult animals. Yet considering that there is no corresponding enhanced sensitivity to heat stress upon knockdown of *pph-4*.*1* or *pph-4*.*2* in adults, this scenario seems unlikely.

Out of all of the other members of the PP2A/4/6 family that we tested, only RNAi targeting *sur-6*, encoding a regulatory subunit of PP2A, reduced the survival of Day 6 adult *C*. *elegans* maintained at elevated temperature (Fig 6E, 6F, 6G and 6H). Knockdown of orthologs of the catalytic, regulatory, or scaffolding subunits of PP2A did not affect the survival of adults challenged by thermal stress (Fig 6H; S9 Fig). Although inhibiting *pph-6* expression increased the resistance of L4 worms the thermal stress (Fig 6C), this effect did not persist into adulthood (Fig 6D). Upon exposure to high temperature, the survival of Day 6 animals treated with RNAi targeting *pph-6* or *saps-1* was comparable to the control group, (Fig 6H; S10 Fig). Our data do not implicate a complete PP2A/4/6 holoenzyme as functioning to confer resistance to heat stress during adulthood. However, individual members of the PP2A/4/6 family may influence the response to thermal stress as part of other pathways in an age-specific manner.

### DAF-16-dependent and -independent functions of members of the PP2A/4/6 subfamily in adult stress resistance

In our functional analysis of orthologs encoding potential subunits of the PP2A/4/6 complexes in *C*. *elegans*, we found a number of genes that, similar to *daf-16*, appear to be important for the resistance to environmental insults during aging since inhibiting their expression through RNAi shortens the survival of adult animals exposed to acute stress. Many of these same genes that encode PP2A or PP4 subunit orthologs are also important for the age-dependent increase in the expression of the DAF-16 transcriptional reporter, *plys-7*::*GFP*, suggesting that their influence on stress resistance may be dependent upon DAF-16. To test this possibility, we revisited our functional analyses, this time targeting genes of interest with RNAi in a *daf-16(mgDf47)* background. We reasoned that if the function of a given gene is indeed dependent upon DAF-16 then knocking down that gene in *daf-16(mgDf47)* mutants should not result in an additive phenotype. This would suggest that the product of the candidate gene and DAF-16 function as part of the same linear pathway. On the other hand, RNAi treatments that modify the survival of *daf-16(mgDf47)* mutants subjected to stress, causing them to die more slowly or more rapidly than untreated *daf-16(mgDf47)* animals, may indicate that the product of the RNAi target functions as part of a separate, parallel pathway.

In this set of experiments each gene encoding a PP2A/PP4 complex member that we found to protect adult wild type animals from bacterial infection, UV irradiation, or heat stress was knocked down in *daf-16(mgDf47)* mutants. At the L4 larval stage and at Day 6 of adulthood *daf-16(mgDf47)* animals subjected to RNAi treatment were challenged with the acute stress to which the targeted genes were found to confer resistance and their survival was measured over time. For consistency, the catalytic subunits of the PP2 and PP4 complexes along with the PP4 regulatory subunit *smk-1* were knocked down in all three environmental stress conditions. As in the wildtype background, the effect of knocking down *let-92* and *paa-1* could only be assessed in adult worms since RNAi had to be initiated at the L4 stage instead of at the L1 stage to avoid aberrant developmental phenotypes.

**Fig 7:**
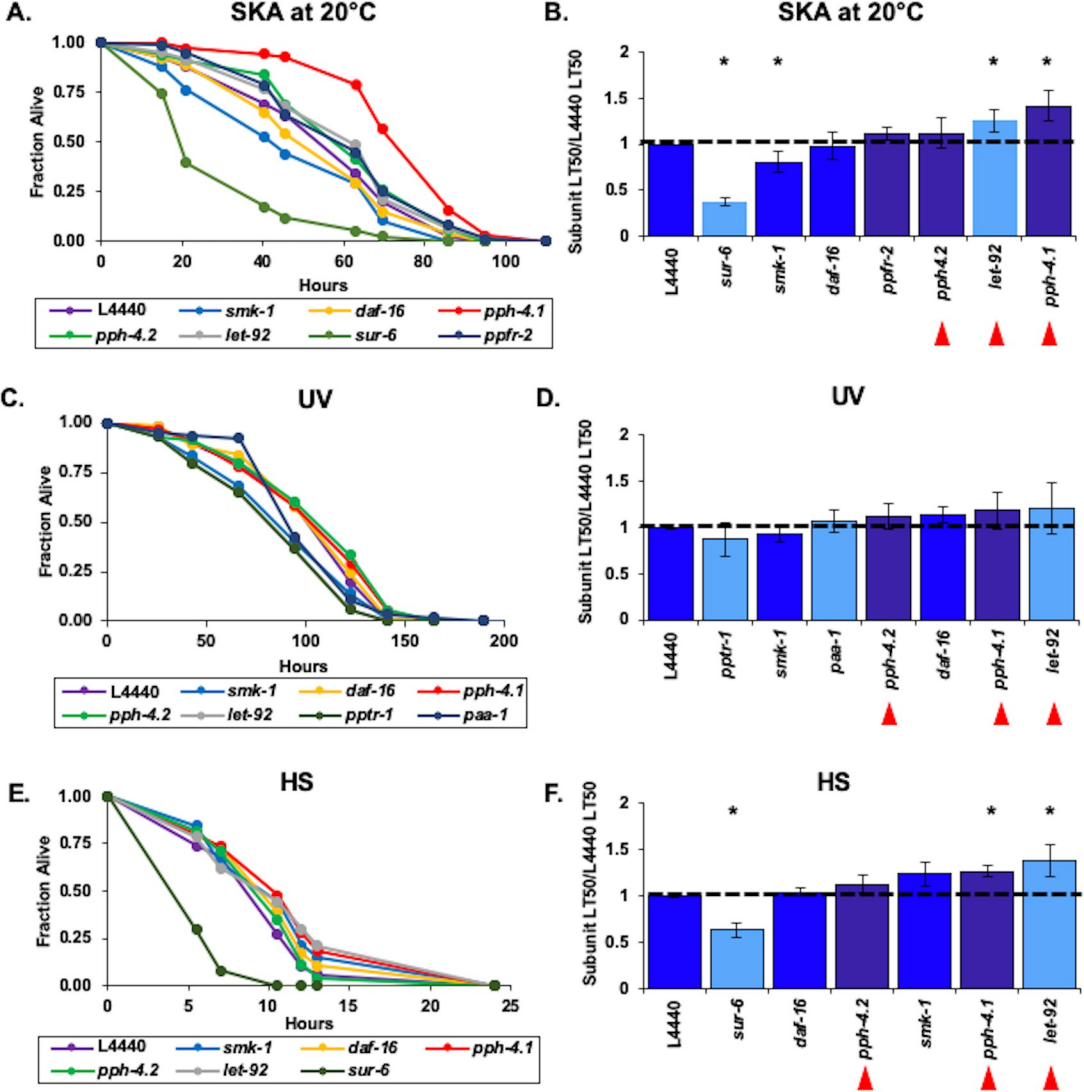
SUR-6, LET-92, and PPH-4.1 may play additional roles in immunity and the response to thermal stress in adult *C*. *elegans* by functioning in pathways that are parallel to DAF-16. Following RNAi treatment beginning at the L1 stage to target *C*. *elegans* homologs of catalytic and selected regulatory subunits of the PP4 and PP2A complexes, *daf-16(mgDf47)* mutant worms were subjected to *P*. *aeruginosa* infection at 20°C (A and B), UV radiation (C and D), or thermal stress at 35˚C (E and F) at D6 of adulthood. The fraction of worms alive at each time point after stress was initiated is plotted as a function of time in hours. In all cases RNAi targeting *daf-16* or *smk-1* and the empty RNAi vector L4440 were included as controls. The average median survival (LT_50_) of animals treated with RNAi targeting the indicated genes following exposure to *P*. *aeruginosa* (B), UV radiation (D), or high temperature (F) at D6 is shown as a fraction of the average median survival of L4440 controls. Bars, standard error of the mean (SEM). Bar colors correspond to the protein phosphatase complex to which products of the indicated genes belong or to controls. Dark blue: L4440, *daf-16*, and *smk-1*; light blue: PP2A; dark purple: PP4. Asterisks indicate RNAi treatments producing statistically significant differences in relative median survival (p<0.05). Horizontal lines are drawn at a relative median survival of 1. Red arrowheads are beneath the names of genes encoding catalytic subunits of the PP2A and 4 complexes.

To improve the resolution of the bacterial infection assay and increase the likelihood of detecting effects of the RNAi treatments on the survival of *daf-16(mgDf47)* animals, *P*. *aeruginosa* infections were conducted at 20˚C instead of 25˚C. Under these conditions, RNAi targeting *smk-1* reproducibly and significantly reduced the ability of L4 *daf-16 (mgDf47)* larvae to resist *P*. *aeruginosa* infection (S11 Fig). Yet knocking down other genes encoding putative subunits of the PP4 complex, including the catalytic subunit *pph-4*.*1* and its paralog *pph-4*.*2*, had no impact on the survival of infected L4 larvae. *smk-1* knockdown also caused Day 6 *daf-16(mgDf47)* mutants to die more rapidly from bacterial infection (Fig 7A and 7B; Table 3). Previously we showed that there is no additive effect between RNAi targeting *smk-1* and a loss-of-function mutation in *daf-16* on the pathogen susceptibility of Day 6 adults [10]. The increase in susceptibility to bacterial infection that we observed here is likely attributable to the results of one of the three biological replicates that were used to calculate the average relative LT_50_. In that particular iteration of the *P*. *aeruginosa* infection assay both the median and maximum lifespan of animals treated with *smk-1* RNAi were uncharacteristically low, as compared to those metrics of survival for *smk-1* RNAi-treated animals in the other two replicates (S12 Fig). RNAi targeting PP4 subunit orthologs *pph-4*.*2* and *ppfr-2* had no effect on the survival of infected Day 6 adult *daf-16(mgDf47)* mutants (Fig 7A and 7B), suggesting that they confer resistance to bacterial infection through the same pathway as *daf-16*. Surprisingly, knockdown of *pph-4*.*1*, the paralog of *pph-4*.*2* encoding the catalytic subunit of PP4, suppressed the enhanced susceptibility phenotype of Day 6 *daf-16 (mgDf47)* mutants as did knockdown of *let-92*, the ortholog of the PP2A catalytic subunit, although to a lesser extent. On the other hand, RNAi targeting *sur-6*, encoding a putative regulatory subunit of the PP2A complex, had the opposite effect and shortened the survival of infected Day 6 *daf-16(mgDf47)* mutants. These data indicate that certain members of the PP2A and PP4 complexes may have functions in adult innate immunity that are independent of DAF-16. Further, they imply that canonical subunits of the same variety of phosphoprotein phosphatase complex may not always act in concert.

**Table 3:** Summary of phenotypes resulting from knockdown of members of the PP2A/4/6 family in *daf-16(mgDf47)* mutants.

		*P*. *aeruginosa* Infection				Heat Stress				UV Irradiation			
		L4		Day 6		L4		Day 6		L4		Day 6	
		Average relative LT50	p-Value	Average relative LT50	p-Value	Average relative LT50	p-Value	Average relative LT50	p-Value	Average relative LT50	p-Value	Average relative LT50	p-Value
	daf-16*	1.029	2.6E-01	0.979	3.9E-01	0.971	1.6E-01	1.039	1.8E-01	0.853	2.1E-01	1.137	8.3E-02
**PP2A**	***let-92***	NA	NA	1.261	**4.9E-02**	NA	NA	1.371	**4.7E-02**	NA	NA	1.203	2.1E-01
	paa-1*	NA	NA	NA	NA	NA	NA	NA	NA	NA	NA	1.061	1.2E-01
	pptr-1*	NA	NA	NA	NA	NA	NA	NA	NA	0.827	9.4E-02	0.868	2.0E-01
	sur-6*	0.932	2.7E-01	0.370	**1.1E-03**	0.951	1.8E-01	0.631	**1.1E-02**	NA	NA	NA	NA
**PP4**	*pph-4*.1*	1.048	1.5E-01	1.419	**3.5E-03**	0.997	4.7E-01	1.261	**1.4E-02**	0.845	2.2E-01	1.183	1.6E-01
	*pph-4*.2*	1.042	1.4E-01	1.120	1.1E-01	1.074	7.3E-02	1.123	1.0E-01	0.892	3.1E-01	1.123	1.6E-01
	smk-1*	0.861	**1.2E-02**	0.803	**1.4E-02**	0.984	2.8E-01	1.231	6.4E-02	0.797	1.5E-01	0.935	2.0E-01
	***ppfr-2***	0.952	7.5E-02	1.119	6.4E-02	NA	NA	NA	NA	NA	NA	NA	NA

* denotes genes that are required for the increase in *Plys-7*::*GFP* expression at Day 6. Average relative LT_50_ was calculated by dividing the average LT_50_ of RNAi-treated animals in a given stress assay by the average LT_50_ of age-matched control animals in that same assay and thus represents the proportional increase or decrease in median lifespan under stressful conditions that are attributable to RNAi inhibition of the gene of interest. The average relative LT_50_ values corresponding to knockdowns that decreased or increased median lifespan after exposure to stress by more than 10% compared to empty vector control are shaded in orange and blue, respectively. Statistical significance was determined by Student’s t-test, with a threshold of p<0.05. p-values associated with significant susceptibility and resistance are bolded.

In wildtype N2 *C*. *elegans*, we found that three genes encoding the subunits to constitute a complete PP2A complex consisting of the catalytic subunit LET-92 along with the regulatory subunit PPTR-1 and the scaffold PAA-1 were required for Day 6 adults to resist ultraviolet irradiation (Fig 5D). *smk-1* was also found to play a role in protecting adult animals from UV light, but it was the only gene encoding a putative member of the PP4 complex found to function in this capacity. None of these four genes has an effect on the resistance of wildtype L4 larvae to UV irradiation (S5 and S6 Figs). We found no evidence of an additive effect on the survival of *C*. *elegans* irradiated as L4 larvae or at Day 6 of adulthood when RNAi was used to target genes encoding PP2A or PP4 subunits in *daf-16(mgDf47)* mutants (S13 Fig; Fig 7C and 7D). In all cases the median lifespan of RNAi-treated animals exposed to UV light was statistically equivalent to that of untreated animals. This result suggests that subunits of the PP2A and PP4 complexes that confer resistance to ultraviolet irradiation do so by functioning in the same pathway as DAF-16.

Of the 20 genes encoding orthologs of PP2A/4/6 complex subunits that we characterized in wildtype animals, only *sur-6* was found to confer thermotolerance to Day 6 adult *C*. *elegans* (Fig 6F and 6H). No other gene encoding a subunit of the PP2A complex, including the catalytic subunit *let-92*, had an effect on those animals to resist exposure to heat stress. In *daf-16(mgDf47)* mutants, knockdown of *sur-6* resulted in an additive phenotype in an age-dependent manner, causing these animals to be more sensitive to heat as compared to mutants treated with the empty RNAi vector L4440 at Day 6 but not at the L4 larval stage. (Fig 7E and 7F; S14 Fig). In contrast, inhibiting expression of *let-92* partially suppressed the enhanced heat sensitivity of *daf-16(mgDf47)* mutants, as did RNAi targeting *pph-4*.*1*. Other RNAi treatments targeting the PP4 complex orthologs *smk-1* and *pph-4*.*2* did not result in any significant change in the survival of heat stressed Day 6 *daf-16(mgDf47)* mutants. Similar to our findings from the bacterial infection assay with *daf-16(mgDf47)* animals, the results of the heat stress experiment suggest that *sur-6*, *let-92*, and *pph-4*.*1* may have *daf-16*-independent functions in parallel pathways that impact the thermotolerance of adult animals.

### Characterization of putative regulators of the PP2A/4/6 family that are not incorporated into the complexes as canonical subunits

**Fig 8:**
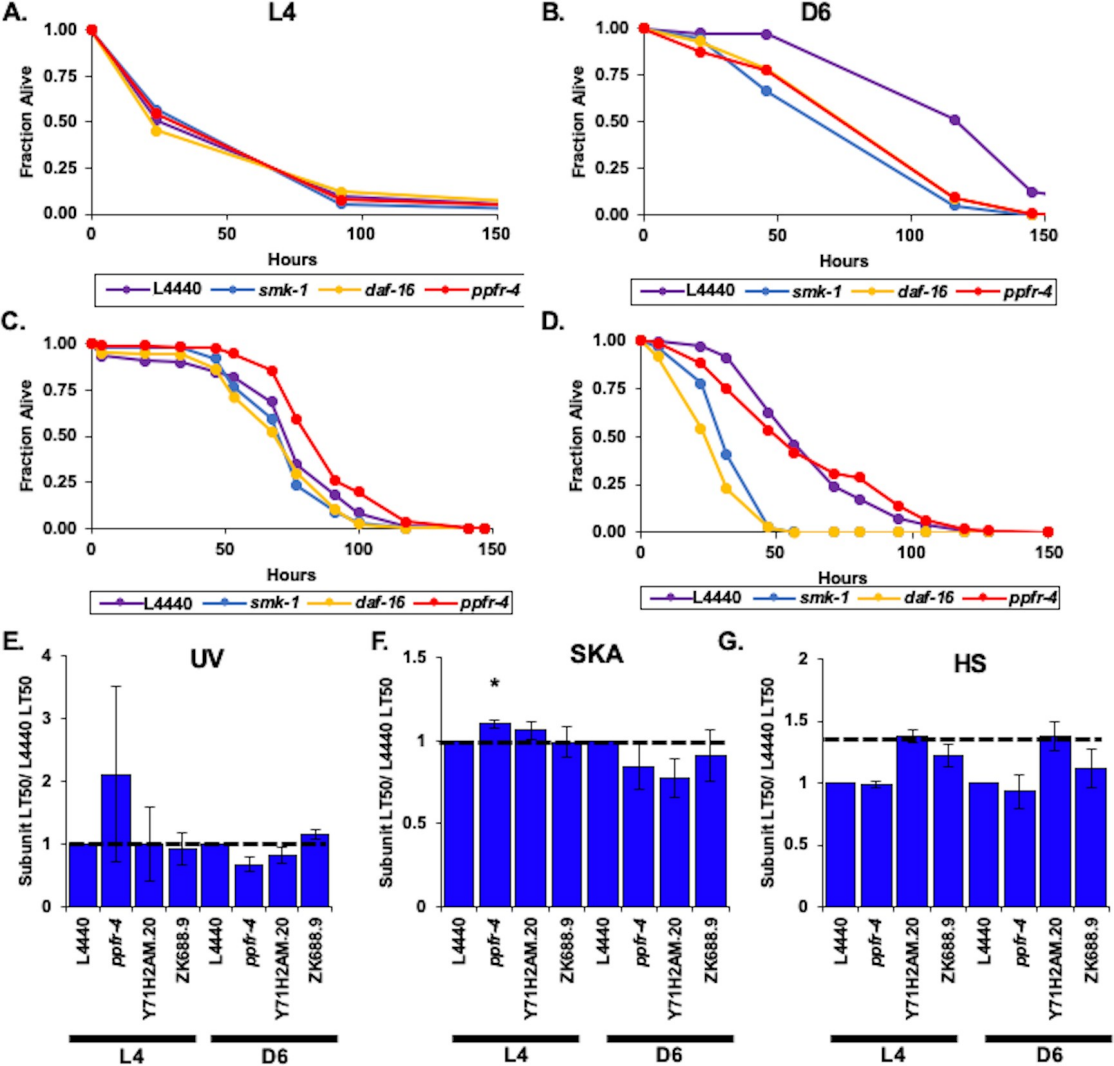
PPFR-4, the *C*. *elegans* ortholog of the ⍺4 regulatory protein, may be required for resistance to UV radiation during adulthood. (A-D) Beginning at the L1 larval stage worms were treated with RNAi to knockdown *ppfr-4*. At the L4 stage (A) or Day 6 of adulthood (B) worms were irradiated with UV light and then returned to standard culture conditions where their survival was monitored over time. The fraction of worms alive at each time point following the UV treatment is plotted as a function of time. RNAi targeting *daf-16* or *smk-1* and the empty RNAi vector L4440 were included as controls. At the L4 stage (C) or Day 6 of adulthood (D) worms were transferred to plates seeded with *P*. *aeruginosa* where their survival was monitored over time. The fraction of worms alive at each time point following the UV treatment is plotted as a function of time. RNAi targeting *daf-16* or *smk-1* and the empty RNAi vector L4440 were included as controls. (E-G) The average relative median survival (LT_50_) of L4 and D6 animals treated with RNAi targeting the indicated genes following exposure to UV light (E), infection with *P*. *aeruginosa* (F) or incubation at 35˚ C (G) is shown as a fraction of the average median survival of L4440 controls. Bars, standard error of the mean (SEM). Asterisks indicate RNAi treatments producing statistically significant differences in median survival (p<0.05). Horizontal lines are drawn at a relative median survival of 1. UV, ultraviolet light; SKA, slow kill assay (*P*. *aeruginosa* infection); HS, heat stress (35˚ C).

In mammals one group of regulatory proteins modulates the function of the entire PP2A/4/6 subfamily of protein phosphatases through mechanisms involving physical interactions and not post-translational modifications. An example of such a protein is α4, which protects the catalytic subunits of the PP2, PP4, and PP6 complexes in humans by inhibiting their ubiquitin-mediated degradation [56]. At the same time, α4 can act as a negative regulator by functioning together with TIPRL. For example, α4 and TIPRL bind noncompetitively to PP2Ac and inactivate it by both expelling metal ions from the active site and displacing the scaffold and regulatory subunits [38]. Conversely, PTPA can reactivate PP2A/4/6 phosphatases by reloading metal ions back in to the active site [53]. Although they are not considered to be constituents of the PP4 holoenzyme, orthologs of all three of these regulators were identified in our database queries as potential interactors of SMK-1/PPH-4.1 (S2 Fig; S2 and S3 Tables). We therefore asked whether they might modulate the PP2A/4/6 family during aging in *C*. *elegans*. Since our data indicate that the PP2A/4/6 family is important for resistance to environmental stress in adult animals, we expected that if PPFR-4 and ZK688.9, the orthologs of α4 and TIPRL, respectively, cooperate to inhibit the phosphatases in *C*. *elegans* as they do in mammals, then knocking them down would increase the survival of Day 6 adults challenged with acute insults. Further, since PTPA acts antagonistically to α4 and TIPRL in mammals, we anticipated that RNAi targeting its ortholog Y71H2AM.20 would cause Day 6 adults to become more sensitive to stress. While knocking down Y71H2AM.20 did increase the susceptibility of Day 6 adults to bacterial infection and their sensitivity to UV light, neither effect was statistically significant (Table 2; Fig 8E and 8F; S15 and S16 Figs). We found no evidence of a role for Y71H2AM.20 in protecting animals from thermal stress (Fig 8G; S17 Fig). Surprisingly, instead of making adult animals more resistant, when *ppfr-4* was targeted by RNAi, Day 6 *C*. *elegans* were more sensitive to UV irradiation, yet the effect on the relative median survival of these animals fell just short of the significance threshold (p = 0.0548) (Fig 8A, 8B and 8E; Table 2). The only resistance phenotype that we found to be associated with *ppfr-4* knockdown was when L4 animals were challenged with *P*. *aeruginosa* but not when adults were infected (Fig 8C, 8D and 8F). Our observations suggest that there may be some differences between the roles of this class of PP2A/4/6 regulators in *C*. *elegans* and mammals, but the lack of statistical significance in our data prevents us from drawing definitive conclusions.

## Discussion

The FoxO transcription factor DAF-16 is a major longevity determinant in *C*. *elegans*. Animals with constitutively active DAF-16 live longer and are more youthful than their wildtype counterparts, suggesting that DAF-16 contributes not only to lifespan but also to healthspan [57]. Accordingly, one category of DAF-16 transcriptional targets are genes that confer resistance to stress, thus shielding animals from long-lasting cellular damage [7]. While exposure to environmental insults induces DAF-16 to upregulate these genes, recent evidence suggests that DAF-16 becomes activated in an age-dependent manner in animals that have not been challenged with a stressor [8, 9]. Since the PP2A/4/6 family of phosphoprotein phosphatases have been implicated in regulating FoxO transcription factors in evolutionarily diverse species [13, 26–29, 31, 33] we asked about their potential role in modulating the activity of DAF-16 in postreproductive adult *C*. *elegans*. In metazoans, the PP2A/4/6 family is especially important at the very early stages of life, ensuring the survival and developmental progression of embryos [19, 58, 59]. In addition, these phosphatases orchestrate the development of multiple tissues including bone, adipose, innate immune cells, and neurons [17–19, 60, 61]. Certain other functions of PP2A/4/6 indicate that later in life they contribute to longevity. For example, PP6 suppresses inflammation and can promote autophagy, both of which would preserve normal lifespan [62, 63]. Regulation of IIS components by PP2A is an example of an even more direct connection between the PP2A/4/6 family and aging, since insulin signaling controls lifespan in invertebrates and other animals [1].

**Fig 9:**
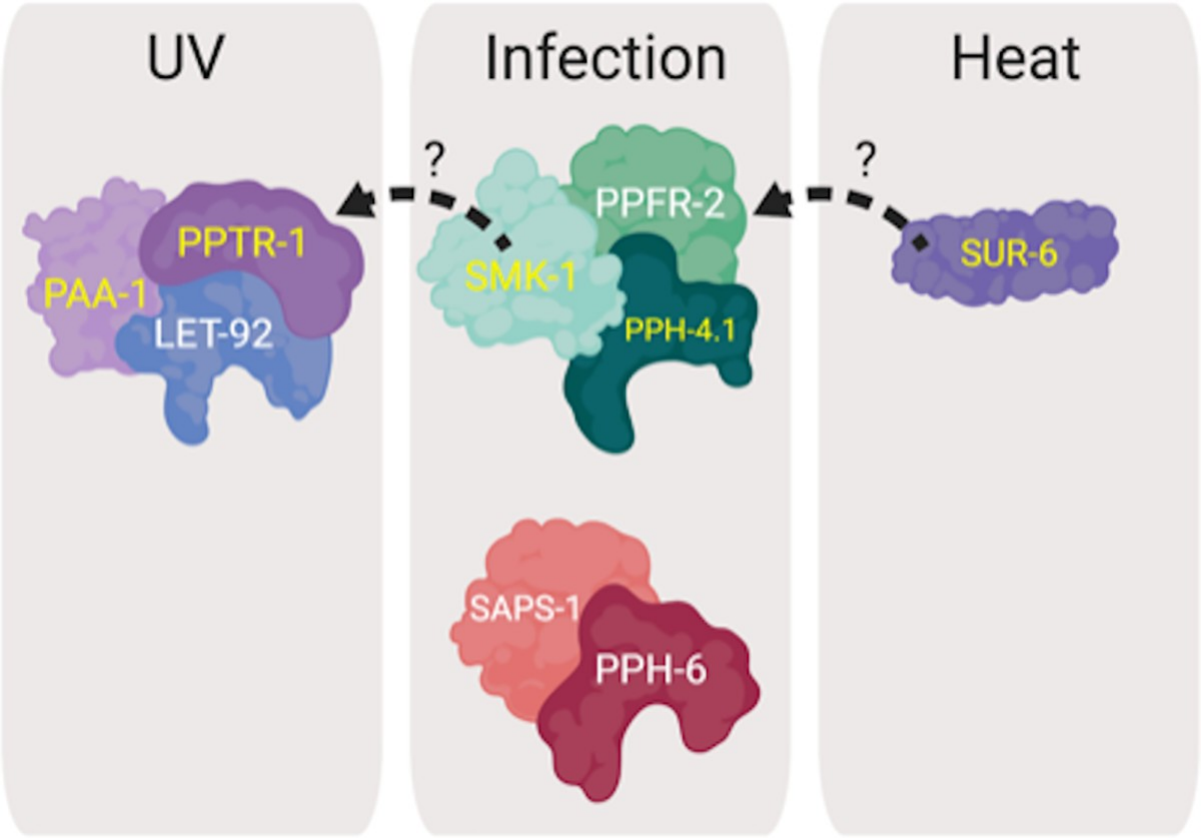
Proposed identity of PP2/4/6 complexes in *C*. *elegans* and their functions during aging. Our data support the existence of at least three central phosphoprotein phosphatase complexes during aging in *C*. *elegans*, but different combinations of subunits allow for more. Different versions of the PP2/4/6 complexes could exist in the same cells simultaneously. Alternatively, the composition of the complexes may be dynamic such that individual regulatory subunits associate and dissociate according to cellular demands or in response to external stimuli. The subunit composition of each complex of the PP2/4/6 family in adult *C*. *elegans* is indicated, according to the following color scheme: purple, PP2A subunits; green, PP4 subunits; red, PP6 subunits. Proteins whose names are labeled in yellow text are required for the age-dependent increase in DAF-16 transcriptional activity as measured in our *in vivo* reporter assay. While the PP2A complex is important for resistance to UV light, both the PP4 and PP6 complexes function in innate immunity in adult worms. Subunit exchange (indicated by dashed arrows) may take place between constituents of the PP2A and PP4 complexes such that SMK-1 associates with a version of the PP2A complex to confer resistance to UV irradiation and SUR-6 associates with PP4 to contribute to host defense. SUR-6 was the only PP2A/4/6 family member found to play a role in thermotolerance during adulthood. Protein structures depicted in this cartoon are for illustrative purposes only.

To investigate the potential role of the PP2A/4/6 phosphatases in regulating DAF-16 during aging in *C*. *elegans*, we used a two-pronged approach where in each branch the phenotype resulting from RNAi knockdown of candidate genes was always compared to the phenotype associated with reduced DAF-16 expression. First we asked whether RNAi-mediated knockdown of genes encoding worm orthologs of human PP2A/4/6 subunits reduced the age-dependent increase in the expression of *plys-7*::*GFP*, an *in vivo* reporter of DAF-16 transcriptional activity. We then challenged RNAi-treated adult animals with three different types of stress to which DAF-16 is known to confer resistance—bacterial infection, heat, and ultraviolet irradiation. Based on this analysis, we identified 9 members of the PP2A/4/6 family that recapitulated *daf-16* knockdown phenotypes in at least one of the functional assays. We found that some groups of candidate genes encoding putative subunits of the same phosphoprotein phosphatase complex also phenocopied each other, with RNAi knockdown resulting in statistically significant enhanced susceptibility to the same environmental stressor. This result was interpreted as evidence of a putative association between the gene products as part of the same protein complex, and it is the basis for the identity of the subunits that we propose as constituents of the phosphoprotein phosphatase complexes that contribute to DAF-16-mediated stress-resistance during adulthood (Fig 9). Further, the constituent subunits are known to be expressed together in the same tissues in *C*. *elegans*, and colocalization to the same subcellular compartment has been experimentally verified for several (S4 Table).

Our data support the possibility of multiple versions of the PP2A/4/6 complexes that are co-expressed during aging in worms that have specialized and partially overlapping functions in preserving health by conferring resistance to environmental insults (Fig 9). First, we found that *let-92*, *paa-1* and *pptr-1* encoding orthologs of the catalytic, scaffolding, and regulatory subunits of the PP2A complex are important for resistance to UV irradiation in Day 6 adult *C*. *elegans*, as is *daf-16*. Our data indicate that *paa-1* and *pptr-1* are necessary for the age-dependent increase in *plys-7*::*GFP* expression suggesting that *paa-1* and *pptr-1* act in the same pathway as *daf-16*. Along those lines, genetic analyses in *daf-16(mgDf47)* mutants suggest that *let-92*, *paa-1*, and *pptr-1* function in the same pathway as *daf-16* to protect adult *C*. *elegans* from UV irradiation. At the same time, they indicate that *let-92* may have an additional function in parallel to *daf-16* to repress both immunity and the response to heat. These functions are likely opposed by DAF-16 since RNAi of *let-92* did not affect the ability of adult wildtype animals to resist either bacterial infection or thermal stress. Our observations both confirm and extend previous investigation of the PP2A complex *C*. *elegans* and in mammals. For example, in *C*. *elegans* PPTR-1 promotes the dephosphorylation of AKT-1 and is required for the extended lifespan and stress resistance phenotypes of *daf-2* mutants [33]. These data strongly implicate PP2A as an indirect DAF-16 regulator in *C*. *elegans*, but no other PP2A complex members were isolated in the screen for *daf-2* suppressors that yielded *pptr-1*. Our data indicate that LET-92 and PAA-1 could complete that tripartite PP2A complex that regulates DAF-16. Supporting this possibility, mammalian orthologues of the same three PP2A subunits that we identified were found to be binding partners of FoxO3a in HEK293 cells [27]. Therefore, although the specific substrates may differ between *C*. *elegans* and mammals, regulation of FoxO transcription factors by PP2A appears to be evolutionarily conserved.

Similar to the effect of inhibiting PPTR-1, loss-of-function mutations in *smk-1*, encoding a regulatory subunit of the PP4 complex, abrogate *daf-2* phenotypes in larvae and young adults, including the elevated expression of DAF-16 transcriptional targets [12]. More recently, RNAi inhibition of the PP4 catalytic subunits *pph-4*.*1* and its paralog *pph*-*4*.*2* were also found to partially suppress *daf-2* [13]. These subunits along with the regulatory subunit PPFR-2 copurify with SMK-1 from wild type, *daf-2(e1370)* and *daf-18 (mg198)* animals, strongly suggesting that they form a complex *in vivo* [13]. Our functional analysis provides independent evidence to suggest this association between PPH-4.1/PPH-4.2, SMK-1 and PPFR-2 to form a PP4 complex in adult *C*. *elegans*. Supporting the possibility that *pph-4*.*1*, *pph-4*.*2* and *smk-1* function in the same pathway as *daf-16*, each is required for the age-dependent increase in *plys-7*::*GFP*. Similar to the effect of knocking down *daf-16*, RNAi targeting *pph-4*.*1*, *pph-4*.*2*, *smk-1*, and *ppfr-2* resulted in an increased susceptibility of wildtype Day 6 adult but not L4 larval stage *C*. *elegans* to infection by *P*. *aeruginosa*. A more severe increase in pathogen susceptibility resulting from *pph-4*.*2* knockdown as compared to *pph-4*.*1* knockdown was the first indication of possible functional divergence between the gene products of these two paralogs. Our functional characterization revealed further phenotypic differences. One is that knocking down *pph-4*.*2* but not *pph-4*.*1* resulted in a mild but statistically insignificant susceptibility to ultraviolet irradiation (Table 2; Fig 5H). Another difference became evident upon knocking down subunits of the PP4 complex in *daf-16(mgDf47)* mutants. While RNAi targeting *pph-4*.*2* did not modify the stress susceptibility phenotypes *daf-16(mgDf47)* mutants in any of our assays, knocking down *pph-4*.*1* suppressed the enhanced susceptibility to pathogen and heat stress of Day 6 *daf-16* animals. This suggests that *pph-4*.*1* but not *pph-4*.*2* acts in parallel to *daf-16* to inhibit innate immunity during adulthood. Considering, however, that *pph-4*.*1* is necessary for the upregulation of *lys-7* during adulthood and that knockdown of *pph-4*.*1* in wildtype animals makes Day 6 adults more susceptible to bacterial infection, this function is likely in addition to its role in modulating the transcriptional activity of DAF-16 and it may, in fact, be antagonized by DAF-16.

Along with its role in innate immunity in Day 6 adult animals, we found that *smk-1* is also important for conferring resistance to ultraviolet radiation (Fig 5). Curiously, it is the only gene encoding a putative subunit of the PP4 complex in *C*. *elegans* that was required for resistance to UV light. RNAi targeting genes encoding other regulatory subunits, *pph-4*.*1* or *pph-4*.*2* had no significant effect on the median lifespan of irradiated adult animals. This observation could be explained by the possibility of subunit exchange between holocomplexes of the PP2A/4/6 family. Said another way, there may be hybrid tripartite holoenzymes where two members that are canonical subunits of one complex (e.g. PP2A) combine with a third member that is considered to be a canonical subunit of a different complex (e.g. PP4). This would require certain regulatory or scaffolding subunits, perhaps including SMK-1, to have the ability to be incorporated into both PP2A and PP4 complexes. Regardless of their specific stoichiometry, biochemical evidence from mammalian systems supports the possibility of interchange of subunits between complexes of the PP2A/4/6 family. In affinity purification or immunoprecipitation experiments in HEK293 cells, PP4c has been isolated as part of complexes with PP2A subunits, including orthologs of PAA-1, SUR-6, and PPTR-2 [37, 46, 47]. Interestingly, in *C*. *elegans* the PP2A scaffold PAA-1 seems to associate with the PP4 subunit SMK-1 [13]. Perhaps to carry out its role in protecting adult worms from exposure from UV light, SMK-1 is incorporated into an alternative version of the LET-92-containing PP2A complex.

Heterotypic associations between members of the PP2A and PP4 complexes could also explain our observation that *sur-6* was the only PP2A subunit whose knockdown resulted in enhanced susceptibility to bacterial infection in Day 6 adult *C*. *elegans*, as did RNAi targeting the PP4 complex members *pph-4*.*1/4*.*2*, *smk-1*, and *ppfr-2* (Fig 4). Unlike the canonical PP4 regulatory subunit *ppfr-2*, we also found *sur-6* to be required for the age-dependent increase in DAF-16 transcriptional activity as measured by the increased expression of the *plys-7*::*GFP* reporter. This implies that there may be a hybrid form of the PPH-4.1/SMK-1 complex that includes SUR-6 as a subunit when modulating DAF-16 transcriptional activity.

Our data suggest additional functions for the PP2A subunit SUR-6 that are independent of DAF-16. RNAi targeting *sur-6* enhanced the susceptibility of Day 6 wildtype *C*. *elegans* to infection by *P*. *aeruginosa* and to thermal stress and it caused Day 6 *daf-16(mgDf47)* mutants to die even more rapidly from both of those insults (Fig 7). This suggests that SUR-6 functions independently of DAF-16 in a parallel pathway, yet the results of our *plys-7*::*GFP* reporter assay indicate that a possible role for SUR-6 in regulating the transcriptional activity of DAF-16 cannot be excluded.

In the course of characterizing the PP2A/4/6 subfamily in our studies, we uncovered a new role for the PP6 complex in innate immunity during aging in *C*. *elegans*. Prior to our work on PPH-6, the *C*. *elegans* ortholog of PP6c, it had only been described in the context of development. In worm embryos PPH-6 is required for spindle positioning, similar to the role of its counterpart PP6c in mammals that controls spindle formation and the condensation, alignment, and segregation of chromosomes during mitosis [64–67]. Based on the other functions of PP6c, we expected that PPH-6 might be necessary to protect adult *C*. *elegans* from ultraviolet irradiation. PP6c functions in both non-homologous end joining (NHEJ) and homology-directed repair of DNA, and without it cells are more sensitive to radiation [68, 69]. Despite the reported role for PP6c, we found no evidence to suggest that either PPH-6 or its regulatory subunit SAPS-1 confer resistance to UV in Day 6 animals. Instead, both *pph-6* and *saps-1* were required for the survival of adult animals challenged with *P*. *aeruginosa*. Neither gene was necessary to increase *plys-7*::*GFP* expression during aging. In light of the nuclear localization of PPH-6 and SAPS-1, however, we speculate that the PP6 complex in *C*. *elegans* could play a role in regulating the expression of immune effectors or detoxifying enzymes to contribute to host defense.

Our work establishes roles for the PP2A, PP4, and PP6 phosphatases in contributing to the healthspan of postreproductive adult *C*. *elegans* through conferring resistance to environmental insults at least in part by modulating transcription. The mechanistic basis for this function will be revealed when the substrates of the complexes in aging animals are uncovered. In light of evidence from mammalian systems, during aging in *C*. *elegans* the PP2A and PP4 complexes may act to dephosphorylate DAF-16 itself or its upstream inhibitory kinases. Yet if this were true and the phosphatases regulate an early step in DAF-16 activation to counteract inhibition through IIS, we may have expected to find significant functional overlap between the PP2A and PP4 complexes such that both would have conferred resistance to the same stresses. Instead, we found that each complex seems to specialize in contributing to the resistance to particular insults. This suggests that PP2A and PP4 may regulate DAF-16 at a more downstream step, possibly functioning to tailor the transcriptional output of DAF-16 to include only discrete subsets of its complete repertoire of targets. Consistent with this possibility, the transcription elongation factor SPT-5 was recently identified as a substrate of PP4 in *daf-2 (e1370)* mutants where it plays a role in recruiting RNA PolII to the promoters of some but not all of the genes regulated by DAF-16 [13]. Should the PP2A/4/6 family act at a similar level in adult wildtype animals, there are other means by which its members are known to influence gene expression that may be of particular relevance to aging. Across evolutionarily diverse species, these protein phosphatases regulate histone modification, chromatin organization, and mRNA processing which all undergo significant changes over time [61, 70–76]. Since we and others find that the PP2A/4/6 phosphatases are important for promoting healthspan, it may be by acting through these pathways that the PP2A/4/6 family modulates the changes in gene expression that are necessary to uphold vitality.

## Supporting information

S1 Fig The catalytic subunits of the PP2A/4/6 subfamily of phosphoprotein phosphatases are highly conserved between *C*. *elegans* and humans. The amino acid sequences of *C*. *elegans* LET-92, PPH-4.1, PPH-4.2 isoform a, and PPH-6 were aligned to their human orthologs PP2Ac, PP4c, and PP6c using Clustal Omega. Residue numbers are indicated on the right. The degree of conservation of individual amino acids across all seven proteins is denoted by symbols where asterisks (*) indicate identical residues, two dots (:) indicate highly similar residues, and one dot (.) indicates somewhat similar residues at a particular position. Characteristic domains of the subfamily are indicated, including the helix switch, loop switch, and TPDYFL motif [36]. Specific residues associated with metal ion coordination and catalysis as well as those that interact with regulatory proteins are denoted by colored symbols according to the legend [36, 39, 40, 77]. In cases where multiple annotations apply to the same residue, corresponding symbols are vertically stacked and squares may be split diagonally.
(TIF)

S2 Fig Interaction network for putative subunits of the *C*. *elegans* PP4 complex reveals possible connections to other members of the PP2A/4/6 subfamily. A list of potential subunits of the *C*. *elegans* PP4 complex including PPFR-1, PPFR-2, PPH-4.1, and SMK-1 in addition to the regulatory protein PPFR-4 was submitted for analysis to Genemania (genemania.org). The resulting network diagram depicts multiple types of interactions including physical interactions (red lines), predicted interactions (orange lines), or genetic interactions (green lines). Other similarities between proteins including co-expression (purple lines) or shared protein domains (yellow lines) are also indicated. In cases where only a Wormbase gene identifier or a CDS identifier was listed in the network diagram, the *C*. *elegans* gene name and/or the human ortholog (in parentheses) is provided in an adjacent grey box. Colored outlines surrounding nodes indicate that a particular protein is either an ortholog of a human PP2A/4/6 complex subunit or is an ortholog of a protein that regulates the activity of one or more members of the PP2A/4/6 family. Since including F46C5.6 as part of the query resulted in a second node that was not part of the larger network it was eliminated from the analysis.
(TIF)

S3 Fig PP4 subunit homologues PPFR-1 and F46C5.6 do not function in innate immunity in *C*. *elegans*. RNAi treatment targeting *C*. *elegans* homologs of regulatory subunits of the PP4 complex was initiated at the L1 stage. After knockdown of F46C5.6 (A,C) or *ppfr-1* (B,D), worms were infected at the L4 larval stage (A, B) or at Day 6 of adulthood (C, D). A representative plot of the fraction of worms alive at each time point after the infection was initiated is shown. In all cases RNAi targeting *daf-16* or *smk-1* and the empty RNAi vector L4440 were included as controls. Statistical analyses indicate that neither of the RNAi treatments had a significant effect on the survival of worms following bacterial infection.
(TIF)

S4 Fig *C*. *elegans* homologues of human PP2A complex subunits with no apparent role in innate immunity during aging. Representative survival curves for worms treated with RNAi to knockdown the indicated gene beginning at L1 and then infected with *P*. *aeruginosa* at the L4 larval stage (A-F) or at Day 6 of adulthood (G-N) are shown. Since RNAi inhibition of *let-92* and *paa-1* arrested larval development, knockdown of those genes was initiated at the L4 stage and worms were infected only at Day 6 (M,N). A representative plot of the fraction of worms alive at each time point after the infection began is shown. All plots include data for animals treated with the empty RNAi vector L4440 and for RNAi knockdown of *daf-16* and *smk-1*. Statistical analyses indicate that none of the RNAi treatments had a significant effect on the survival of worms following bacterial infection.
(TIF)

S5 Fig Three regulatory subunits of the PP4 complex do not appear to function in conferring resistance to UV irradiation. RNAi treatment targeting *C*. *elegans* homologs of regulatory subunits of the PP4 complex F46C5.6 (A, D), *ppfr-1* (B, E), and *ppfr-2* (C, F) was initiated at the L1 stage and continued for the duration of the assay. Worms were exposed to UV irradiation at the L4 larval stage (A-C) or at D6 of adulthood (D-F) after which their survival under standard culturing conditions was monitored. A representative plot of the fraction of worms alive at each time point following exposure to UV radiation is shown. All plots include data for animals treated with the empty RNAi vector L4440 and for RNAi knockdown of *daf-16* and *smk-1*. Statistical analyses indicate that none of the RNAi treatments had a significant effect on the survival of worms following UV irradiation.
(TIF)

S6 Fig *C*. *elegans* homologues of human PP2A complex subunits with no apparent role in conferring resistance to UV irradiation. Representative survival curves for worms treated with RNAi to knockdown the indicated gene beginning at L1 and irradiated with ultraviolet light at the L4 larval stage (A-F) or at Day 6 of adulthood (G-L) are shown. Following irradiation, worms were returned to standard culture conditions and their survival was monitored over time. A representative plot of the fraction of worms alive at each time point following exposure to UV radiation is shown. All plots include data for animals treated with the empty RNAi vector L4440 and for RNAi knockdown of *daf-16* and *smk-1*. Statistical analyses indicate that none of the RNAi treatments had a significant effect on the survival of worms following UV irradiation.
(TIF)

S7 Fig The PP6 regulatory subunit SAPS-1 plays no role in protecting *C*. *elegans* from UV irradiation. RNAi treatment targeting *C*. *elegans* homolog of the PP6 regulatory subunit *saps-1* was initiated at the L1 stage and continued for the duration of the assay. Worms were exposed to UV irradiation at the L4 larval stage (A) or at D6 of adulthood (B) after which their survival under standard culturing conditions was monitored. A representative plot of the fraction of worms alive at each time point following exposure to UV radiation is shown. All plots include data for animals treated with the empty RNAi vector L4440 and for RNAi knockdown of *daf-16* and *smk-1*. Statistical analyses indicate that RNAi targeting *saps-1* had no significant effect on the survival of worms following UV irradiation.
(TIF)

S8 Fig PP4 subunit homologues PPFR-1 and PPFR-2 do not confer thermotolerance to *C*. *elegans*. From L1 until death *C*. *elegans* were treated with RNAi targeting putative regulatory subunits of the PP4 complex *ppfr-1* (A, C) or *ppfr-2* (B,D). Worms were shifted from 20˚ C to 35˚ C at larval stage L4 (A, B) or D6 (C, D) and maintained at the high temperature until death. A representative plot of the fraction of worms alive at each time point during the incubation at 35˚ C is shown. In all cases RNAi targeting *daf-16* or *smk-1* and the empty RNAi vector L4440 were included as controls. Statistical analyses indicate that neither of the RNAi treatments had a significant effect on the survival of worms under heat stress.
(TIF)

S9 Fig *C*. *elegans* homologues of human PP2A complex subunits with no apparent role in thermotolerance during adulthood. From L1 until death worms were treated with RNAi targeting *C*. *elegans* orthologs of catalytic and regulatory subunits of the human PP2A complex. At either the L4 stage (A-F) or at Day 6 of adulthood (G-N) worms were shifted from the standard incubation temperature of 20˚ C to 35˚ C and were maintained at the high temperature until death. Since RNAi inhibition of *let-92* and *paa-1* arrested larval development, knockdown of those genes was initiated at the L4 stage and worms were shifted to high temperature only at Day 6 (M,N). A representative plot of the fraction of worms alive at each time point during the incubation at 35˚ C is shown. In all cases RNAi targeting *daf-16* or *smk-1* and the empty RNAi vector L4440 were included as controls. Statistical analyses indicate that none of the RNAi treatments had a significant effect on the survival of worms under heat stress.
(TIF)

S10 Fig The PP6 regulatory subunit SAPS-1 plays no role in the response to heat stress. From L1 until death *C*. *elegans* were treated with RNAi targeting *saps-1*, a subunit of the PP6 complex in *C*. *elegans*. Worms were shifted from 20˚ C to 35˚ C at larval stage L4 (A) or D6 (B) and maintained at the high temperature until death. A representative plot of the fraction of worms alive at each time point during the incubation at 35˚ C is shown. In all cases RNAi targeting *daf-16* or *smk-1* and the empty RNAi vector L4440 were included as controls. Statistical analyses indicate that RNAi targeting *saps-1* did not have a significant effect on the survival of worms under heat stress.
(TIF)

S11 Fig SMK-1, but not other putative members of the PP2A or PP4 complexes, contributes to *C*. *elegans* larval immunity independently of DAF-16. At the L4 larval stage *daf-16(mgdf47)* worms that had been treated with RNAi targeting homologs of catalytic and selected regulatory subunits of the PP4 and PP2A complexes since the L1 stage were infected with *P*. *aeruginosa* (A-F). The results from four independent biological replicates probing the function of orthologs of subunits of the PP4 complex (A-D) and three biological replicates probing the function of the PP2A subunit SUR-6 (D-F) in comparison to PP4 subunits are shown. In each replicate RNAi targeting *daf-16* or *smk-1* and the empty RNAi vector L4440 were included as controls. The fraction of worms alive at each time point after infection was initiated is plotted as a function of time in hours. The average relative median survival (LT_50_) of animals treated with RNAi targeting the indicated genes following stress with *P*. *aeruginosa* at L4 is shown as a fraction of the average median survival of L4440 controls (G). Bars, standard error of the mean (SEM). Bar colors correspond to the protein phosphatase complex to which products of the indicated genes belong or to controls. Dark blue: L4440, *daf-16*, and *smk-1*; light blue: PP2A; dark purple: PP4. Asterisks indicate RNAi treatments producing statistically significant differences in relative median survival (p<0.05). A dashed horizontal line indicates relative median survival of 1. Red arrowheads are beneath the names of genes encoding catalytic subunits of the PP4 complex.
(TIF)

S12 Fig Additional replicates of stress assays with RNAi-treated Day 6 *daf-16(mgDf47)* mutants. RNAi treatment to target the indicated *C*. *elegans* homologs of catalytic and selected regulatory subunits of the PP4 and PP2A complexes was initiated at the L1 larval stage in *daf-16(mgDf47)* mutants. At Day 6 of adulthood these animals were subjected to *P*. *aeruginosa* infection at 20°C (A and B), UV radiation (C and D), or heat stress at 35˚C (E and F). Adjacent panels show data from independent biological replicates. The fraction of worms alive after stress was initiated was measured at regular intervals and is plotted as a function of time in hours. In all cases RNAi targeting *daf-16* or *smk-1* and the empty RNAi vector L4440 were included as controls. All replicates depicted here contribute to the averages depicted in Fig 7B, 7D and 7F.
(TIF)

S13 Fig *C*. *elegans* orthologs of selected PP2A and PP4 complex members do not affect susceptibility to UV radiation at L4 in the absence of *daf-16*. *daf-16(mgDf47)* L4 larvae that had been treated with RNAi starting at the L1 stage to target selected regulatory subunits of the PP4 and PP2A complexes were briefly exposed to UV radiation and their survival was then measured over time (A-C). The fraction of worms alive at each time point after radiation is plotted as a function of time in hours. The results from three independent biological replicates are shown. In all cases RNAi targeting *daf-16* or *smk-1* and the empty RNAi vector L4440 were included as controls. The average relative median survival (LT_50_) of animals treated with RNAi targeting the indicated genes following UV irradiation at L4 is shown as a fraction of the average median survival of L4440 controls (D). Bars, standard error of the mean (SEM). Bar colors correspond to the protein phosphatase complex to which products of the indicated genes belong or to controls. Dark blue: L4440, *daf-16*, and *smk-1*; light blue: PP2A; dark purple: PP4. The dashed horizontal line denotes a relative median survival of 1. Red arrowheads are beneath the names of genes encoding catalytic subunits of the PP4 complex.
(TIF)

S14 Fig *C*. *elegans* orthologs of selected PP2A and PP4 complex members play no additional role in thermotolerance in *daf-16(mgDf47)* larvae. RNAi treatment targeting homologues of catalytic and selected regulatory subunits of the PP4 and PP2A complexes was initiated in L1 stage *daf-16(mgDf47)* larvae which were then subjected to heat stress at 35˚C at the L4 stage (A-C). The fraction of worms alive at each time point after infection was initiated is plotted as a function of time in hours. The results from three independent biological replicates are shown. In all cases RNAi targeting *daf-16* or *smk-1* and the empty RNAi vector L4440 were included as controls. The average relative median survival (LT_50_) of animals treated with RNAi targeting the indicated genes following initiation of heat stress (D) at L4 is shown as a fraction of the average median survival of L4440 controls. Bars, standard error of the mean (SEM). Bar colors correspond to the protein phosphatase complex to which products of the indicated genes belong or to controls. Dark blue: L4440, *daf-16*, and *smk-1*; light blue: PP2A; dark purple: PP4. The horizontal line is drawn at a relative median survival of 1. Red arrowheads are beneath the names of genes encoding catalytic subunits of the PP4 complex.
(TIF)

S15 Fig *C*. *elegans* orthologs of regulators of the PP2A/4/6 family do not contribute to host defense during adulthood. Beginning at the L1 stage the *C*. *elegans* homologs of human PTPA (Y71H2AM.20; A, C) and TIPRL (ZK688.9; B, D) were targeted with RNAi. Worms were infected with *P*. *aeruginosa* at the L4 (A, B) larval stage or at D6 (C, D) of adulthood. A representative plot of the fraction of worms alive at each time point after the infection was initiated is shown. In all cases RNAi targeting *daf-16* or *smk-1* and the empty RNAi vector L4440 were included as controls. Statistical analyses indicate that RNAi treatments targeting Y71H2AM.20 and ZK688.9 had no significant effect on the survival of worms following bacterial infection.
(TIF)

S16 Fig *C*. *elegans* orthologs of PTPA and TIPRL have no apparent function in conferring resistance to UV irradiation. RNAi treatment targeting the PTPA ortholog Y71H2AM.20 (A, B) or the TIPRL ortholog ZK688.9 (C, D) was initiated at the L1 stage and continued for the duration of the assay. Worms were exposed to UV irradiation at the L4 larval stage (A, C) or at D6 of adulthood (B, D) after which their survival under standard culturing conditions was monitored. A representative plot of the fraction of worms alive at each time point following exposure to UV radiation is shown. All plots include data for animals treated with the empty RNAi vector L4440 and for RNAi knockdown of *daf-16* and *smk-1*. Statistical analyses indicate that RNAi targeting Y71H2AM.20 or ZK688.9 had no significant effect on the survival of worms following UV irradiation.
(TIF)

S17 Fig *C*. *elegans* orthologs of regulators of the PP2/4/6 family do not affect susceptibility to heat stress. Beginning at the L1 stage the *C*. *elegans* homologs of human ⍺4 (*ppfr-4*; A, D), PTPA (Y71H2AM.20; B, E), and TIPRL (ZK688.9; C, F) were targeted with RNAi. Worms were shifted from 20˚ C to 35˚ C at larval stage L4 (A-C) or D6 (D-F) and maintained at the high temperature until death. A representative plot of the fraction of worms alive at each time point during the incubation at 35˚ C is shown. In all cases RNAi targeting *daf-16* or *smk-1* and the empty RNAi vector L4440 were included as controls. Statistical analyses indicate that RNAi treatments targeting *ppfr-4*, Y71H2AM.20, and ZK688.9 had no significant effect on the survival of worms under heat stress.
(TIF)

S1 Table Percent identity matrix for *C*. *elegans* and human PP2A/4/6 catalytic subunits. Output from Clustal Omega alignment of *C*. *elegans* and human catalytic subunits of the PP2A/4/6 subfamily showing percent identity between each pairwise comparison. The best matching ortholog in each column (highest percent identity) is highlighted in green.
(XLSX)

S2 Table PPH-4.1 interactors. Predicted and experimentally validated interactors of PPH-4.1 as listed in the Wormbase protein interaction database are shown.
(XLSX)

S3 Table SMK-1 interactors. Predicted and experimentally validated interactors of SMK-1 as listed in the Wormbase protein interaction database are shown.
(XLSX)

S4 Table Location and timing of expression of *C*. *elegans* PP2A/4/6 genes. Genes listed in this table correspond to the following three groups: 1) those encoding members of the holoenzyme complexes that we propose in Fig 9; 2) T22D1.5 and F43B10.1, orthologs of PP2B” regulatory subunits that we found to be necessary for the age-dependent increase in DAF-16 transcriptional activity; and 3) orthologs of regulatory proteins. *daf-16* expression information is included as a reference. Expression of genes in tissues where DAF-16 function has been explicitly tested in previous studies, including body wall muscle, intestine, and neurons, are highlighted by coloring the names of those tissues (red, green, and blue, respectively) in the “Anatomical sites of expression” column.
(XLSX)
