# Supplementary figures and images for: The PP2A/4/6 subfamily of phosphoprotein phosphatases regulates DAF-16 and confers resistance to environmental stress in postreproductive adult C. elegans
Source: PLoS One. 2020 Dec 14;15(12):e0229812. doi: 10.1371/journal.pone.0229812 (PMC7735605; doi:10.1371/journal.pone.0229812)

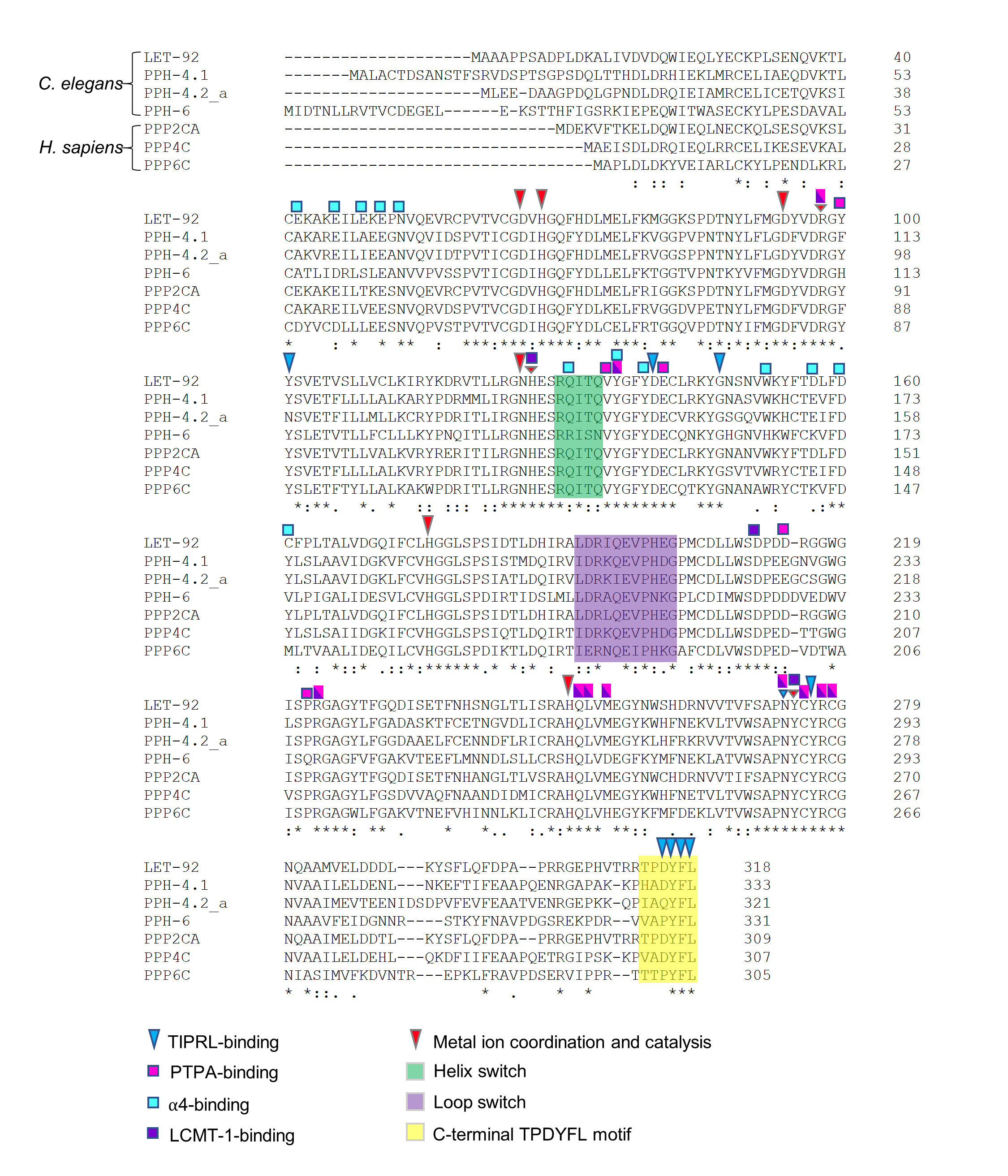

Supplement: S1 Fig — The amino acid sequences of C. elegans LET-92, PPH-4.1, PPH-4.2 isoform a, and PPH-6 were aligned to their human orthologs PP2Ac, PP4c, and PP6c using Clustal Omega. Residue numbers are indicated on the right. The degree of conservation of individual amino acids across all seven proteins is denoted by symbols where asterisks (*) indicate identical residues, two dots (:) indicate highly similar residues, and one dot (.) indicates somewhat similar residues at a particular position. Characteristic domains of the subfamily are indicated, including the helix switch, loop switch, and TPDYFL motif [36]. Specific residues associated with metal ion coordination and catalysis as well as those that interact with regulatory proteins are denoted by colored symbols according to the legend [36, 39, 40, 77]. In cases where multiple annotations apply to the same residue, corresponding symbols are vertically stacked and squares may be split diagonally. (TIF) [file pone.0229812.s001.tif]

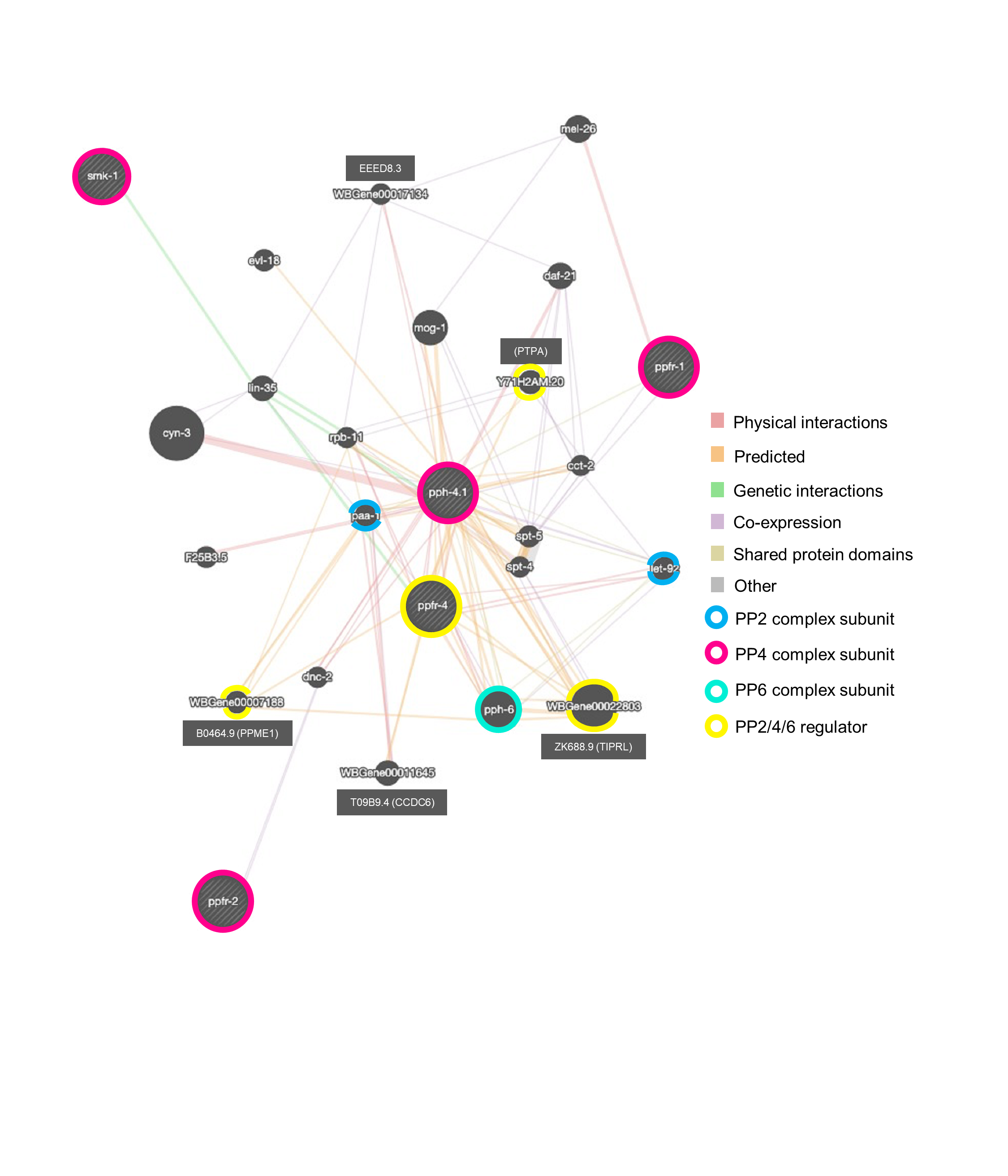

Supplement: S2 Fig — A list of potential subunits of the C. elegans PP4 complex including PPFR-1, PPFR-2, PPH-4.1, and SMK-1 in addition to the regulatory protein PPFR-4 was submitted for analysis to Genemania (genemania.org). The resulting network diagram depicts multiple types of interactions including physical interactions (red lines), predicted interactions (orange lines), or genetic interactions (green lines). Other similarities between proteins including co-expression (purple lines) or shared protein domains (yellow lines) are also indicated. In cases where only a Wormbase gene identifier or a CDS identifier was listed in the network diagram, the C. elegans gene name and/or the human ortholog (in parentheses) is provided in an adjacent grey box. Colored outlines surrounding nodes indicate that a particular protein is either an ortholog of a human PP2A/4/6 complex subunit or is an ortholog of a protein that regulates the activity of one or more members of the PP2A/4/6 family. Since including F46C5.6 as part of the query resulted in a second node that was not part of the larger network it was eliminated from the analysis. (TIF) [file pone.0229812.s002.tif]

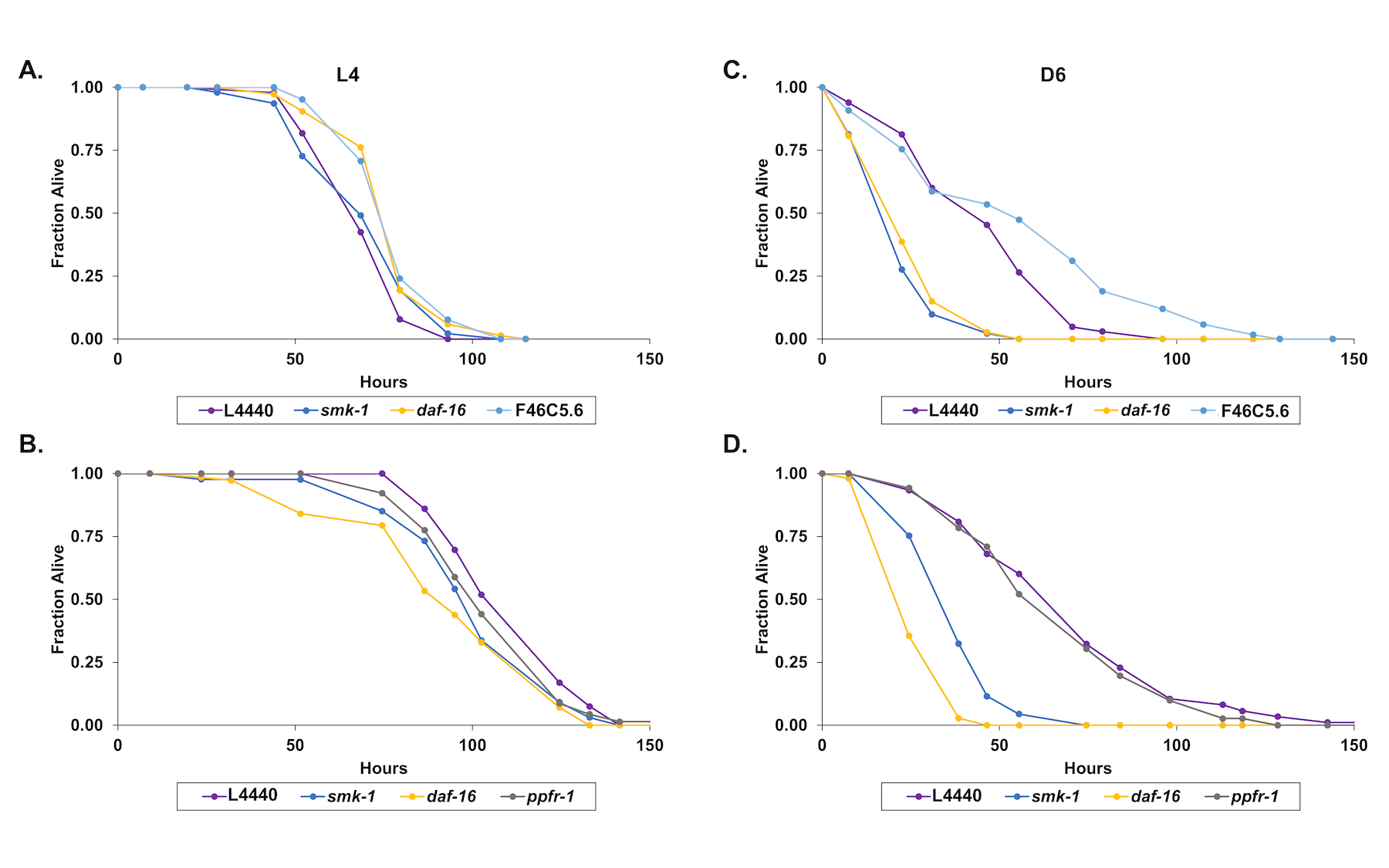

Supplement: S3 Fig — RNAi treatment targeting C. elegans homologs of regulatory subunits of the PP4 complex was initiated at the L1 stage. After knockdown of F46C5.6 (A,C) or ppfr-1 (B,D), worms were infected at the L4 larval stage (A, B) or at Day 6 of adulthood (C, D). A representative plot of the fraction of worms alive at each time point after the infection was initiated is shown. In all cases RNAi targeting daf-16 or smk-1 and the empty RNAi vector L4440 were included as controls. Statistical analyses indicate that neither of the RNAi treatments had a significant effect on the survival of worms following bacterial infection. (TIF) [file pone.0229812.s003.tif]

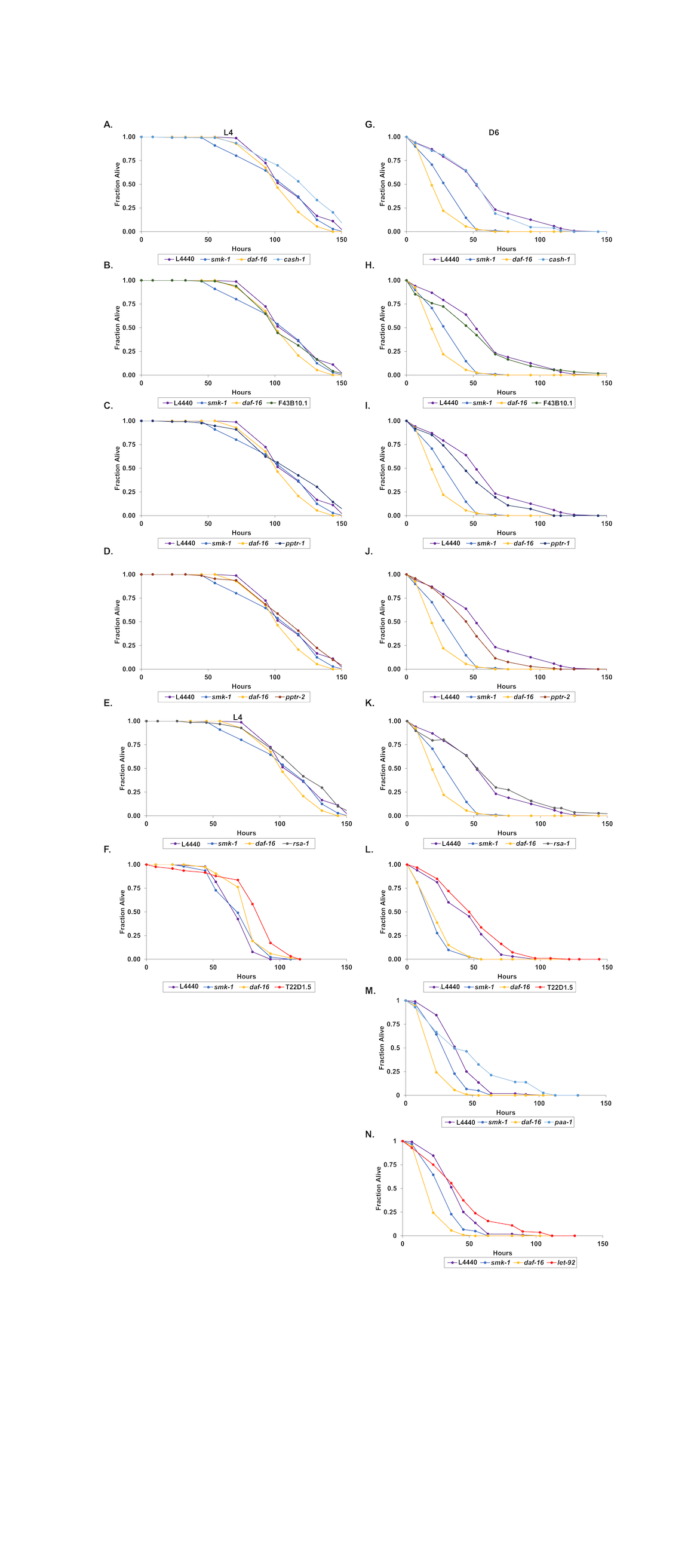

Supplement: S4 Fig — Representative survival curves for worms treated with RNAi to knockdown the indicated gene beginning at L1 and then infected with P. aeruginosa at the L4 larval stage (A-F) or at Day 6 of adulthood (G-N) are shown. Since RNAi inhibition of let-92 and paa-1 arrested larval development, knockdown of those genes was initiated at the L4 stage and worms were infected only at Day 6 (M,N). A representative plot of the fraction of worms alive at each time point after the infection began is shown. All plots include data for animals treated with the empty RNAi vector L4440 and for RNAi knockdown of daf-16 and smk-1. Statistical analyses indicate that none of the RNAi treatments had a significant effect on the survival of worms following bacterial infection. (TIF) [file pone.0229812.s004.tif]

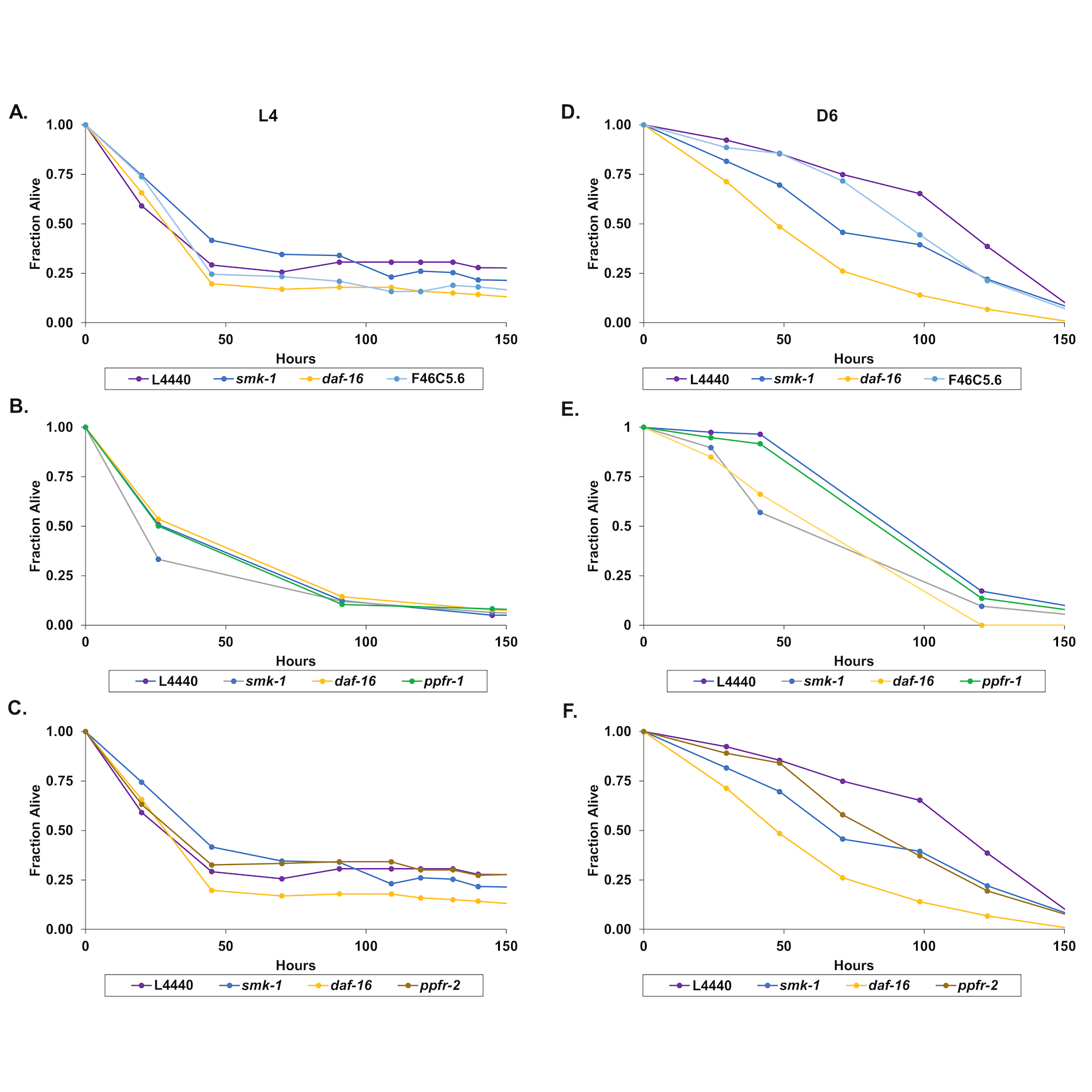

Supplement: S5 Fig — RNAi treatment targeting C. elegans homologs of regulatory subunits of the PP4 complex F46C5.6 (A, D), ppfr-1 (B, E), and ppfr-2 (C, F) was initiated at the L1 stage and continued for the duration of the assay. Worms were exposed to UV irradiation at the L4 larval stage (A-C) or at D6 of adulthood (D-F) after which their survival under standard culturing conditions was monitored. A representative plot of the fraction of worms alive at each time point following exposure to UV radiation is shown. All plots include data for animals treated with the empty RNAi vector L4440 and for RNAi knockdown of daf-16 and smk-1. Statistical analyses indicate that none of the RNAi treatments had a significant effect on the survival of worms following UV irradiation. (TIF) [file pone.0229812.s005.tif]

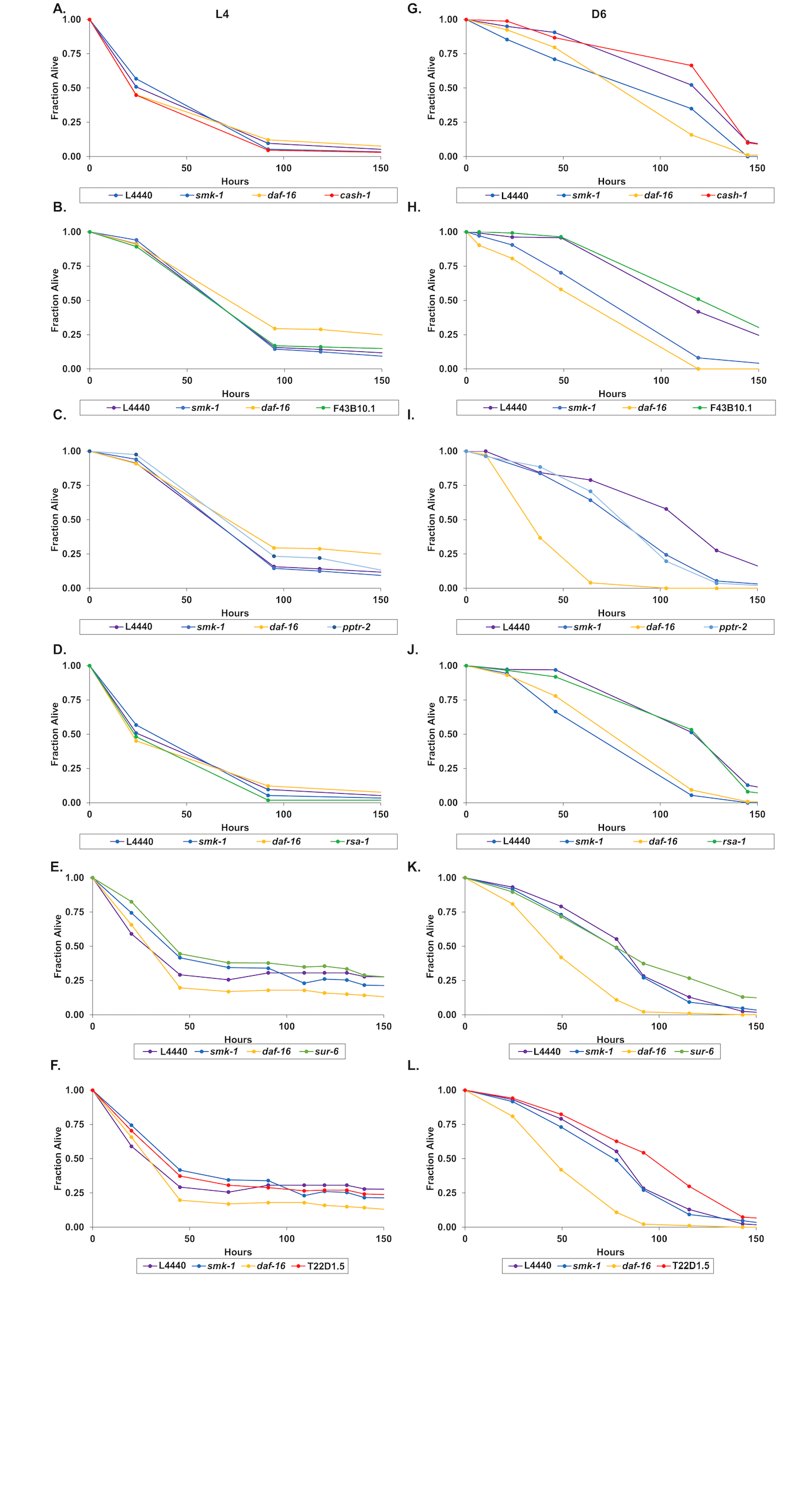

Supplement: S6 Fig — Representative survival curves for worms treated with RNAi to knockdown the indicated gene beginning at L1 and irradiated with ultraviolet light at the L4 larval stage (A-F) or at Day 6 of adulthood (G-L) are shown. Following irradiation, worms were returned to standard culture conditions and their survival was monitored over time. A representative plot of the fraction of worms alive at each time point following exposure to UV radiation is shown. All plots include data for animals treated with the empty RNAi vector L4440 and for RNAi knockdown of daf-16 and smk-1. Statistical analyses indicate that none of the RNAi treatments had a significant effect on the survival of worms following UV irradiation. (TIF) [file pone.0229812.s006.tif]

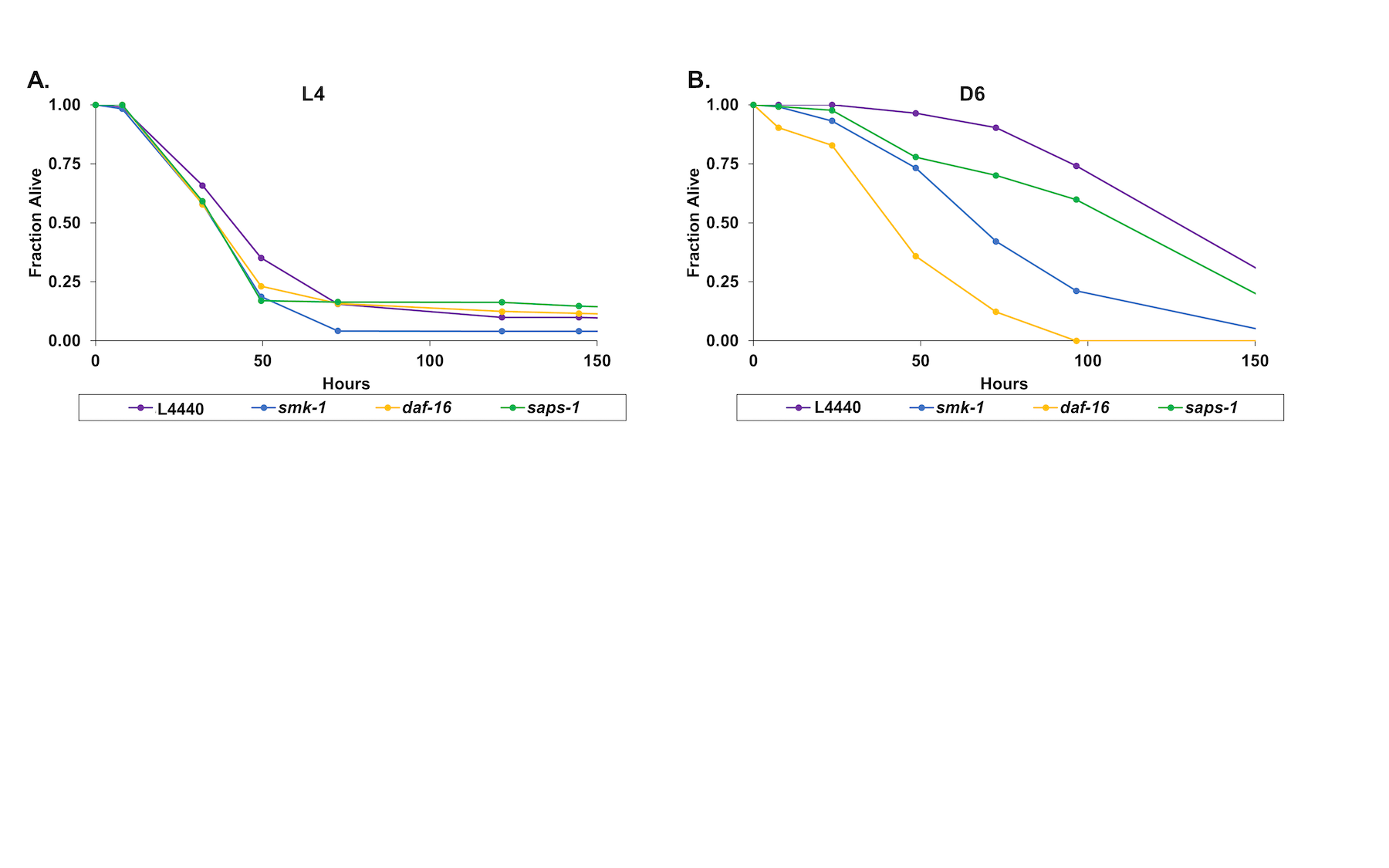

Supplement: S7 Fig — RNAi treatment targeting C. elegans homolog of the PP6 regulatory subunit saps-1 was initiated at the L1 stage and continued for the duration of the assay. Worms were exposed to UV irradiation at the L4 larval stage (A) or at D6 of adulthood (B) after which their survival under standard culturing conditions was monitored. A representative plot of the fraction of worms alive at each time point following exposure to UV radiation is shown. All plots include data for animals treated with the empty RNAi vector L4440 and for RNAi knockdown of daf-16 and smk-1. Statistical analyses indicate that RNAi targeting saps-1 had no significant effect on the survival of worms following UV irradiation. (TIF) [file pone.0229812.s007.tif]

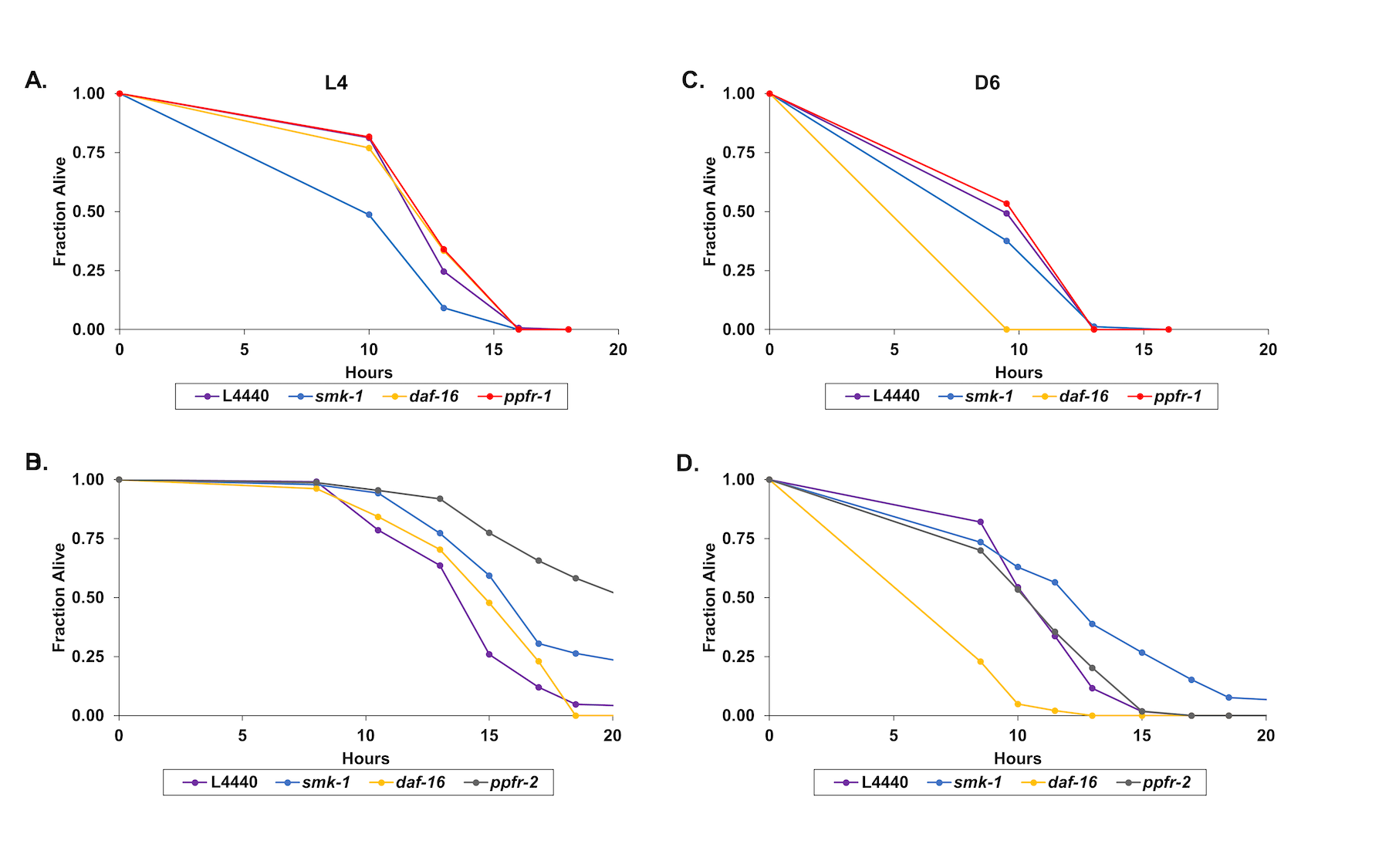

Supplement: S8 Fig — From L1 until death C. elegans were treated with RNAi targeting putative regulatory subunits of the PP4 complex ppfr-1 (A, C) or ppfr-2 (B,D). Worms were shifted from 20˚ C to 35˚ C at larval stage L4 (A, B) or D6 (C, D) and maintained at the high temperature until death. A representative plot of the fraction of worms alive at each time point during the incubation at 35˚ C is shown. In all cases RNAi targeting daf-16 or smk-1 and the empty RNAi vector L4440 were included as controls. Statistical analyses indicate that neither of the RNAi treatments had a significant effect on the survival of worms under heat stress. (TIF) [file pone.0229812.s008.tif]

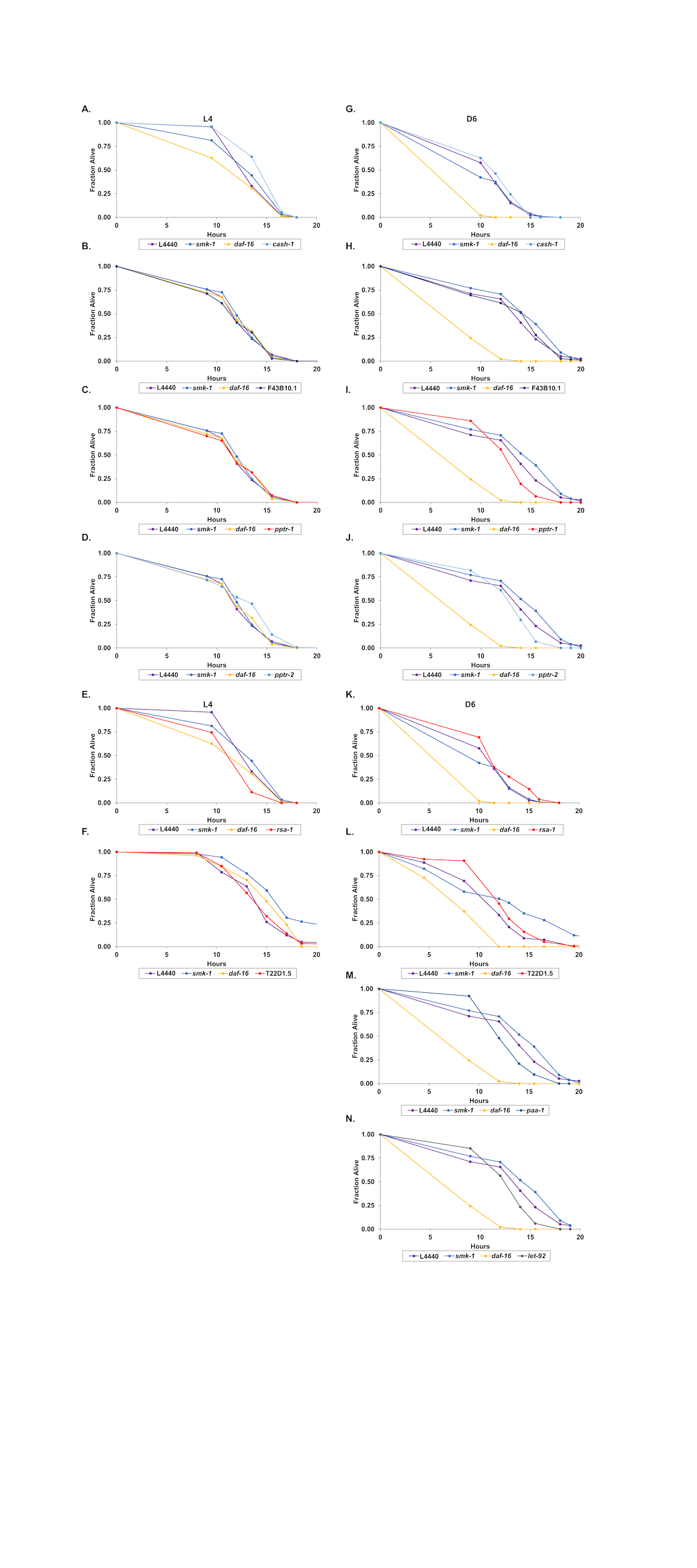

Supplement: S9 Fig — From L1 until death worms were treated with RNAi targeting C. elegans orthologs of catalytic and regulatory subunits of the human PP2A complex. At either the L4 stage (A-F) or at Day 6 of adulthood (G-N) worms were shifted from the standard incubation temperature of 20˚ C to 35˚ C and were maintained at the high temperature until death. Since RNAi inhibition of let-92 and paa-1 arrested larval development, knockdown of those genes was initiated at the L4 stage and worms were shifted to high temperature only at Day 6 (M,N). A representative plot of the fraction of worms alive at each time point during the incubation at 35˚ C is shown. In all cases RNAi targeting daf-16 or smk-1 and the empty RNAi vector L4440 were included as controls. Statistical analyses indicate that none of the RNAi treatments had a significant effect on the survival of worms under heat stress. (TIF) [file pone.0229812.s009.tif]

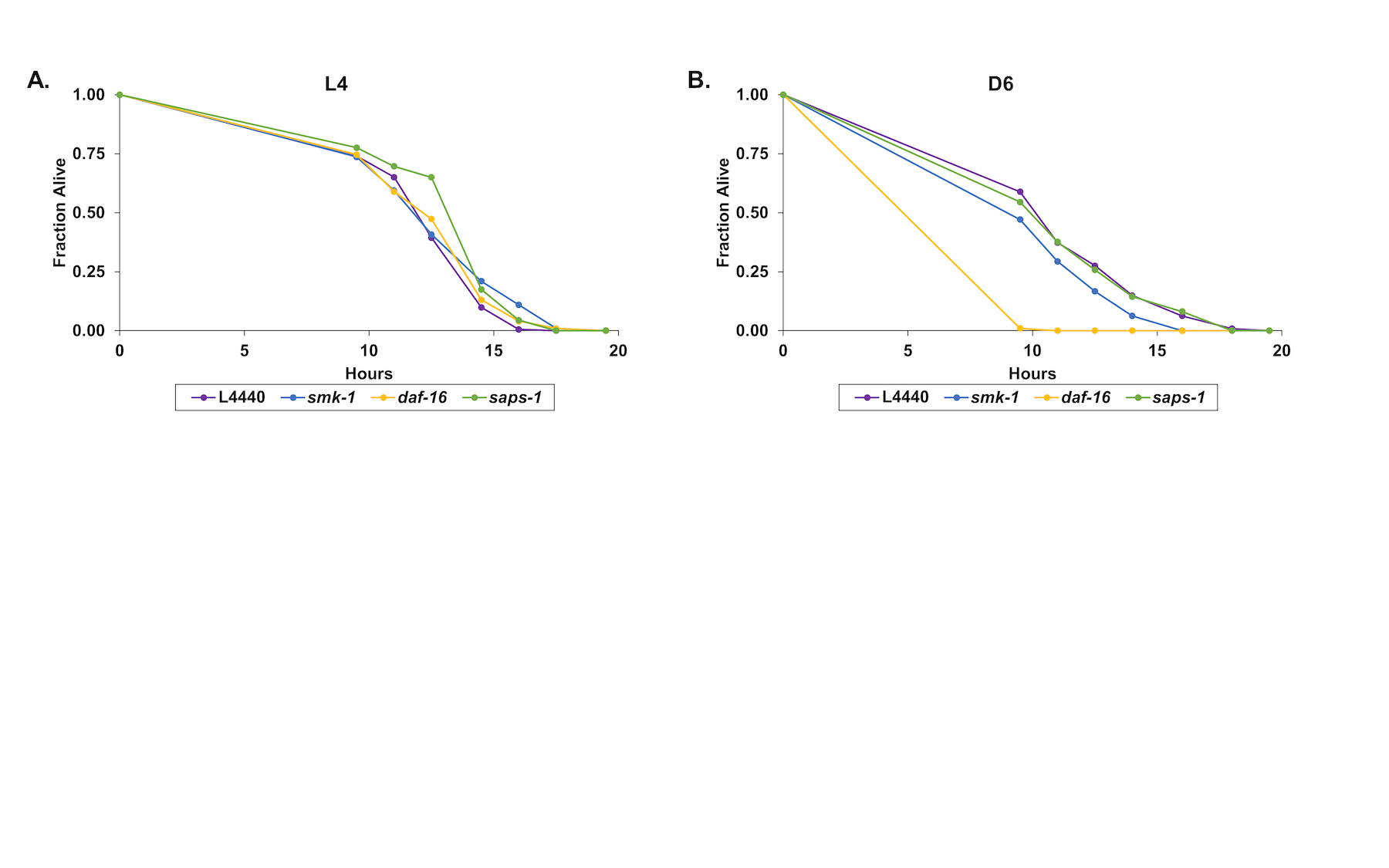

Supplement: S10 Fig — From L1 until death C. elegans were treated with RNAi targeting saps-1, a subunit of the PP6 complex in C. elegans. Worms were shifted from 20˚ C to 35˚ C at larval stage L4 (A) or D6 (B) and maintained at the high temperature until death. A representative plot of the fraction of worms alive at each time point during the incubation at 35˚ C is shown. In all cases RNAi targeting daf-16 or smk-1 and the empty RNAi vector L4440 were included as controls. Statistical analyses indicate that RNAi targeting saps-1 did not have a significant effect on the survival of worms under heat stress. (TIF) [file pone.0229812.s010.tif]

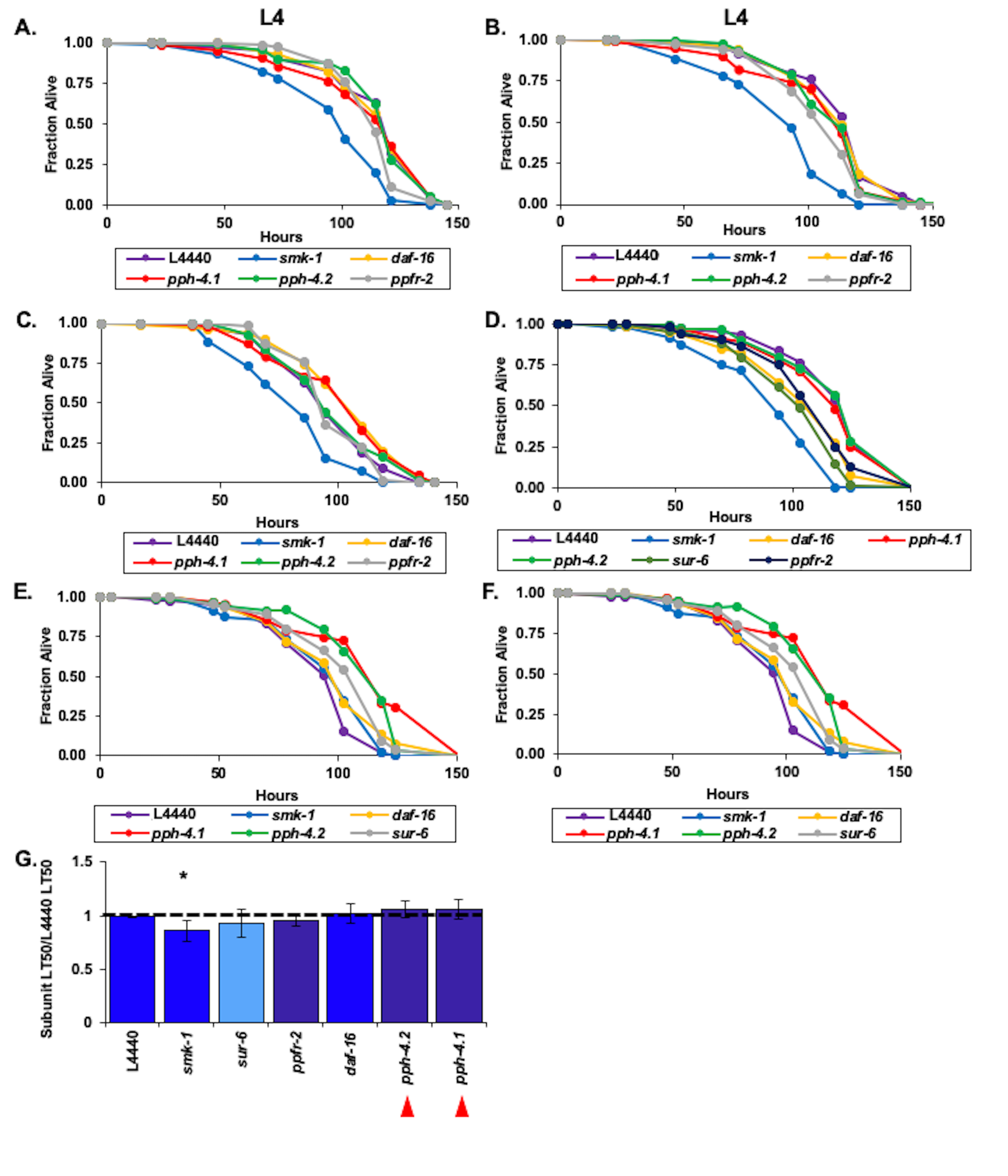

Supplement: S11 Fig — At the L4 larval stage daf-16(mgdf47) worms that had been treated with RNAi targeting homologs of catalytic and selected regulatory subunits of the PP4 and PP2A complexes since the L1 stage were infected with P. aeruginosa (A-F). The results from four independent biological replicates probing the function of orthologs of subunits of the PP4 complex (A-D) and three biological replicates probing the function of the PP2A subunit SUR-6 (D-F) in comparison to PP4 subunits are shown. In each replicate RNAi targeting daf-16 or smk-1 and the empty RNAi vector L4440 were included as controls. The fraction of worms alive at each time point after infection was initiated is plotted as a function of time in hours. The average relative median survival (LT50) of animals treated with RNAi targeting the indicated genes following stress with P. aeruginosa at L4 is shown as a fraction of the average median survival of L4440 controls (G). Bars, standard error of the mean (SEM). Bar colors correspond to the protein phosphatase complex to which products of the indicated genes belong or to controls. Dark blue: L4440, daf-16, and smk-1; light blue: PP2A; dark purple: PP4. Asterisks indicate RNAi treatments producing statistically significant differences in relative median survival (p<0.05). A dashed horizontal line indicates relative median survival of 1. Red arrowheads are beneath the names of genes encoding catalytic subunits of the PP4 complex. (TIF) [file pone.0229812.s011.tif]

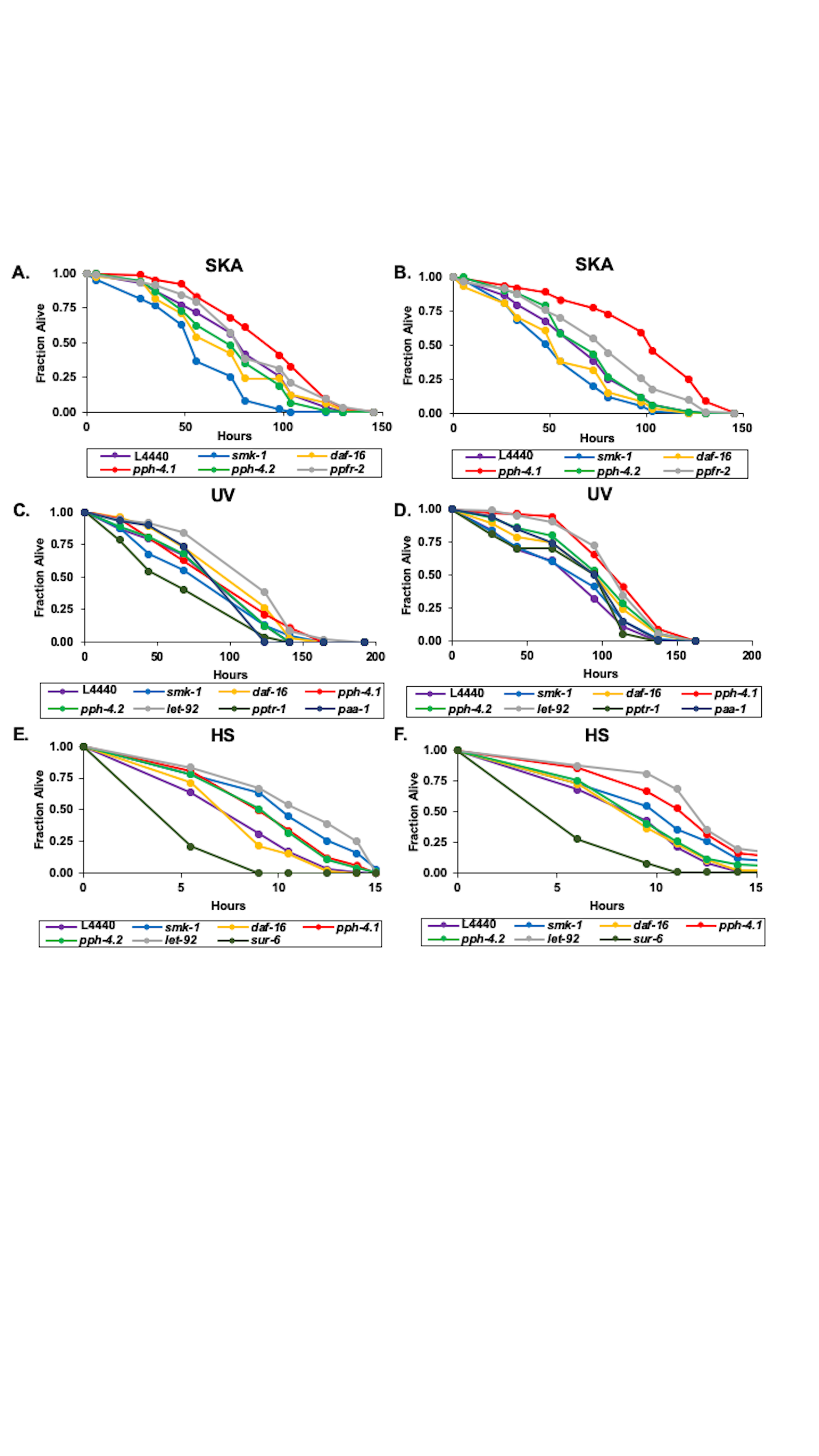

Supplement: S12 Fig — RNAi treatment to target the indicated C. elegans homologs of catalytic and selected regulatory subunits of the PP4 and PP2A complexes was initiated at the L1 larval stage in daf-16(mgDf47) mutants. At Day 6 of adulthood these animals were subjected to P. aeruginosa infection at 20°C (A and B), UV radiation (C and D), or heat stress at 35˚C (E and F). Adjacent panels show data from independent biological replicates. The fraction of worms alive after stress was initiated was measured at regular intervals and is plotted as a function of time in hours. In all cases RNAi targeting daf-16 or smk-1 and the empty RNAi vector L4440 were included as controls. All replicates depicted here contribute to the averages depicted in Fig 7B, 7D and 7F. (TIF) [file pone.0229812.s012.tif]

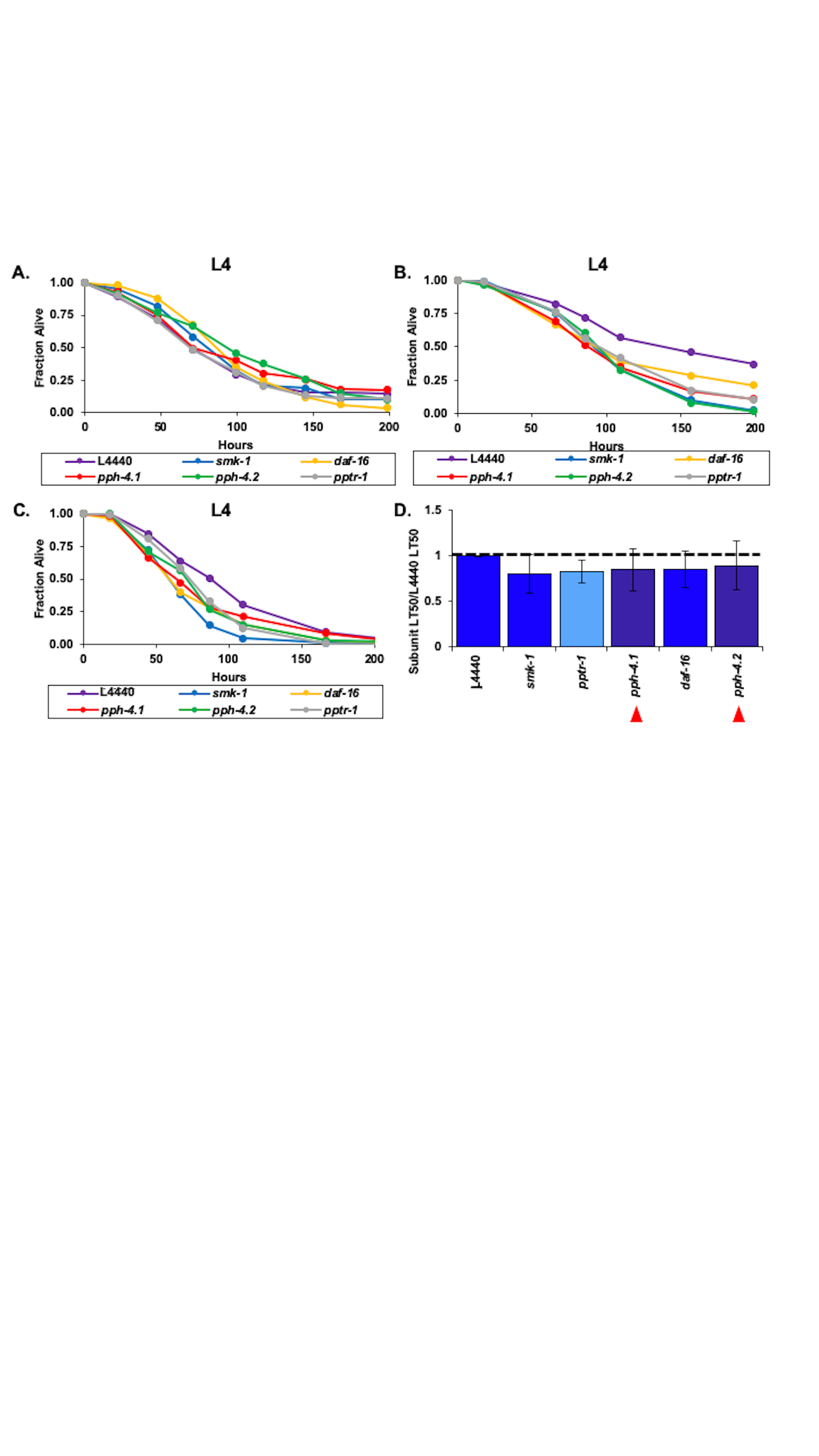

Supplement: S13 Fig — daf-16(mgDf47) L4 larvae that had been treated with RNAi starting at the L1 stage to target selected regulatory subunits of the PP4 and PP2A complexes were briefly exposed to UV radiation and their survival was then measured over time (A-C). The fraction of worms alive at each time point after radiation is plotted as a function of time in hours. The results from three independent biological replicates are shown. In all cases RNAi targeting daf-16 or smk-1 and the empty RNAi vector L4440 were included as controls. The average relative median survival (LT50) of animals treated with RNAi targeting the indicated genes following UV irradiation at L4 is shown as a fraction of the average median survival of L4440 controls (D). Bars, standard error of the mean (SEM). Bar colors correspond to the protein phosphatase complex to which products of the indicated genes belong or to controls. Dark blue: L4440, daf-16, and smk-1; light blue: PP2A; dark purple: PP4. The dashed horizontal line denotes a relative median survival of 1. Red arrowheads are beneath the names of genes encoding catalytic subunits of the PP4 complex. (TIF) [file pone.0229812.s013.tif]

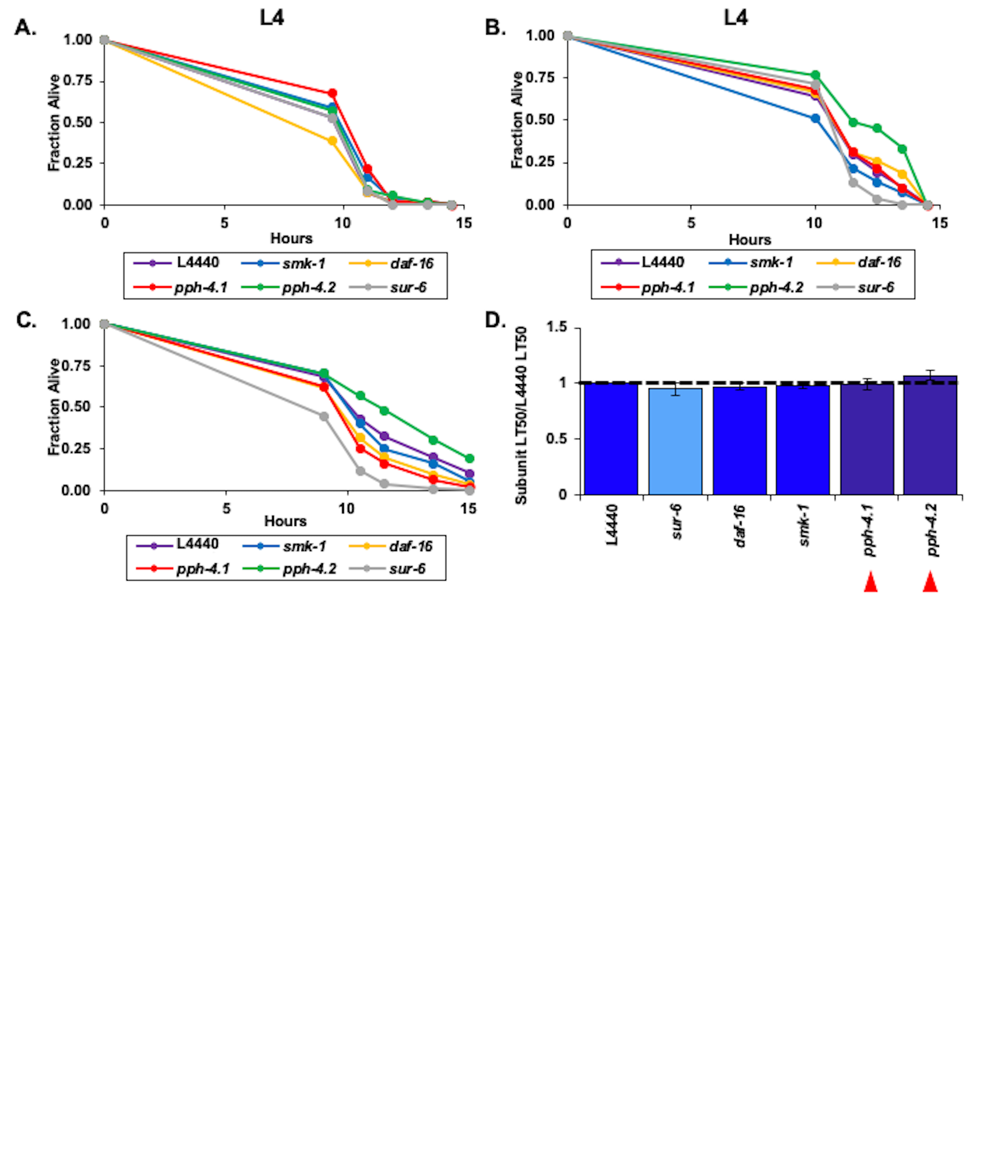

Supplement: S14 Fig — RNAi treatment targeting homologues of catalytic and selected regulatory subunits of the PP4 and PP2A complexes was initiated in L1 stage daf-16(mgDf47) larvae which were then subjected to heat stress at 35˚C at the L4 stage (A-C). The fraction of worms alive at each time point after infection was initiated is plotted as a function of time in hours. The results from three independent biological replicates are shown. In all cases RNAi targeting daf-16 or smk-1 and the empty RNAi vector L4440 were included as controls. The average relative median survival (LT50) of animals treated with RNAi targeting the indicated genes following initiation of heat stress (D) at L4 is shown as a fraction of the average median survival of L4440 controls. Bars, standard error of the mean (SEM). Bar colors correspond to the protein phosphatase complex to which products of the indicated genes belong or to controls. Dark blue: L4440, daf-16, and smk-1; light blue: PP2A; dark purple: PP4. The horizontal line is drawn at a relative median survival of 1. Red arrowheads are beneath the names of genes encoding catalytic subunits of the PP4 complex. (TIF) [file pone.0229812.s014.tif]

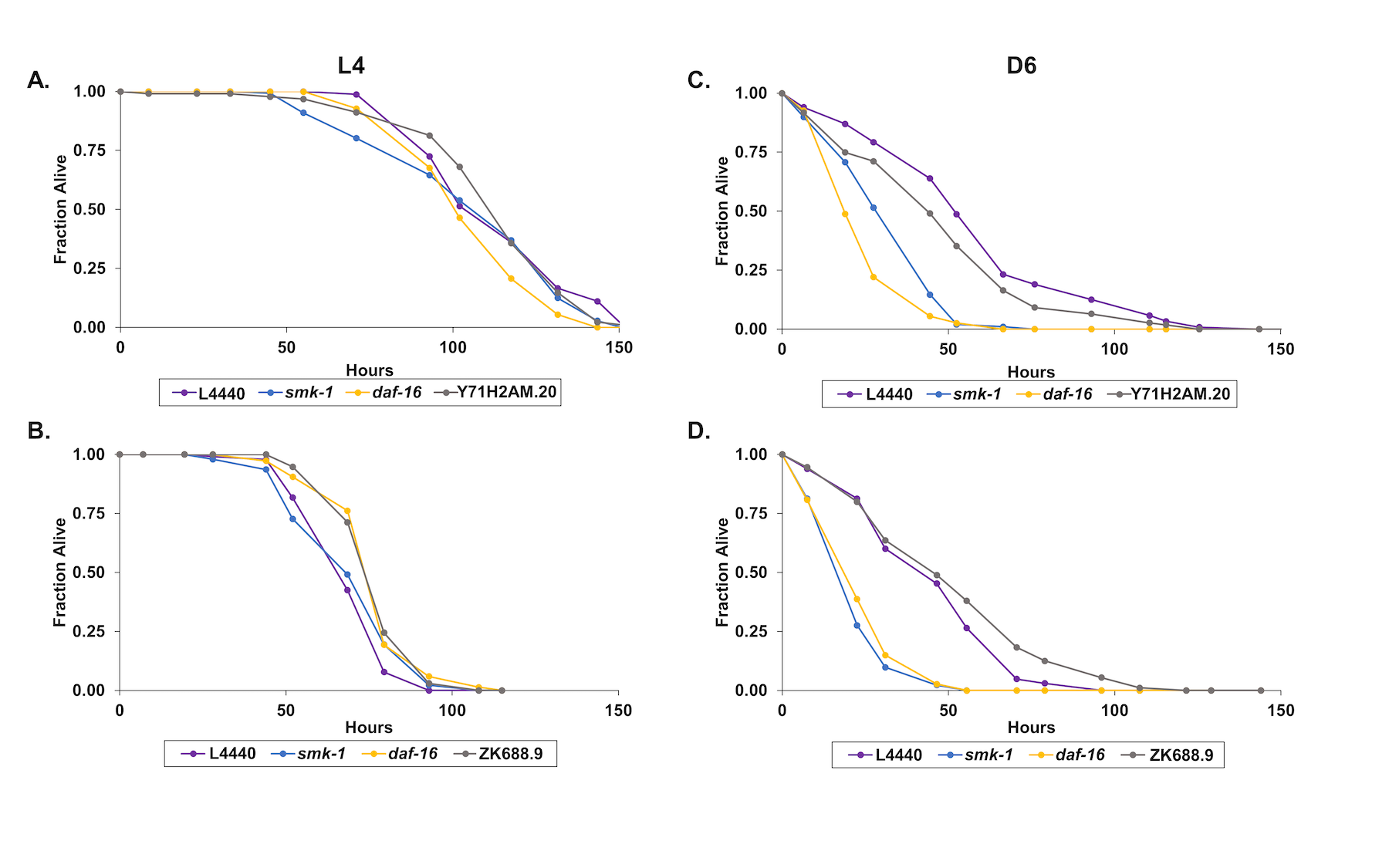

Supplement: S15 Fig — Beginning at the L1 stage the C. elegans homologs of human PTPA (Y71H2AM.20; A, C) and TIPRL (ZK688.9; B, D) were targeted with RNAi. Worms were infected with P. aeruginosa at the L4 (A, B) larval stage or at D6 (C, D) of adulthood. A representative plot of the fraction of worms alive at each time point after the infection was initiated is shown. In all cases RNAi targeting daf-16 or smk-1 and the empty RNAi vector L4440 were included as controls. Statistical analyses indicate that RNAi treatments targeting Y71H2AM.20 and ZK688.9 had no significant effect on the survival of worms following bacterial infection. (TIF) [file pone.0229812.s015.tif]

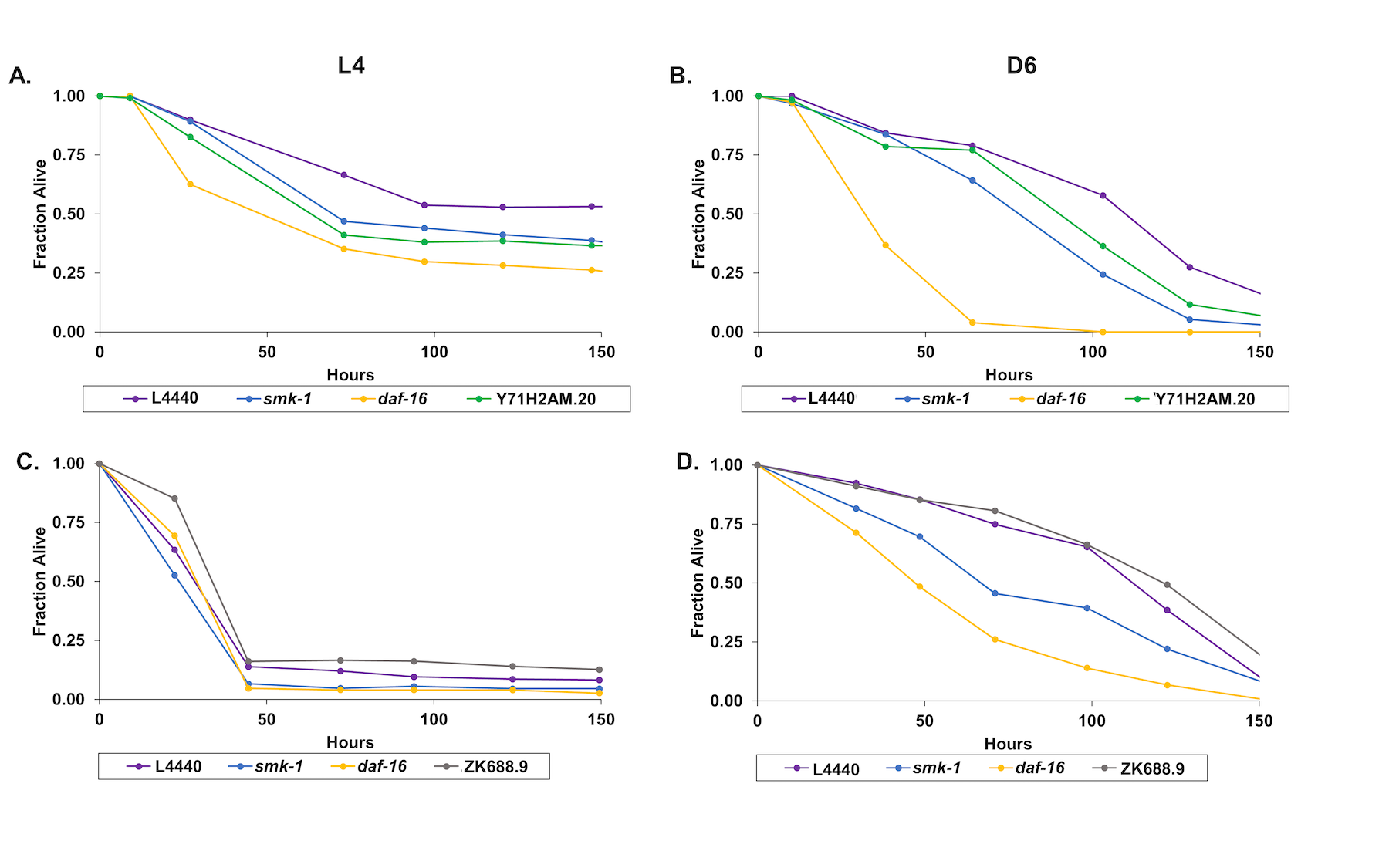

Supplement: S16 Fig — RNAi treatment targeting the PTPA ortholog Y71H2AM.20 (A, B) or the TIPRL ortholog ZK688.9 (C, D) was initiated at the L1 stage and continued for the duration of the assay. Worms were exposed to UV irradiation at the L4 larval stage (A, C) or at D6 of adulthood (B, D) after which their survival under standard culturing conditions was monitored. A representative plot of the fraction of worms alive at each time point following exposure to UV radiation is shown. All plots include data for animals treated with the empty RNAi vector L4440 and for RNAi knockdown of daf-16 and smk-1. Statistical analyses indicate that RNAi targeting Y71H2AM.20 or ZK688.9 had no significant effect on the survival of worms following UV irradiation. (TIF) [file pone.0229812.s016.tif]

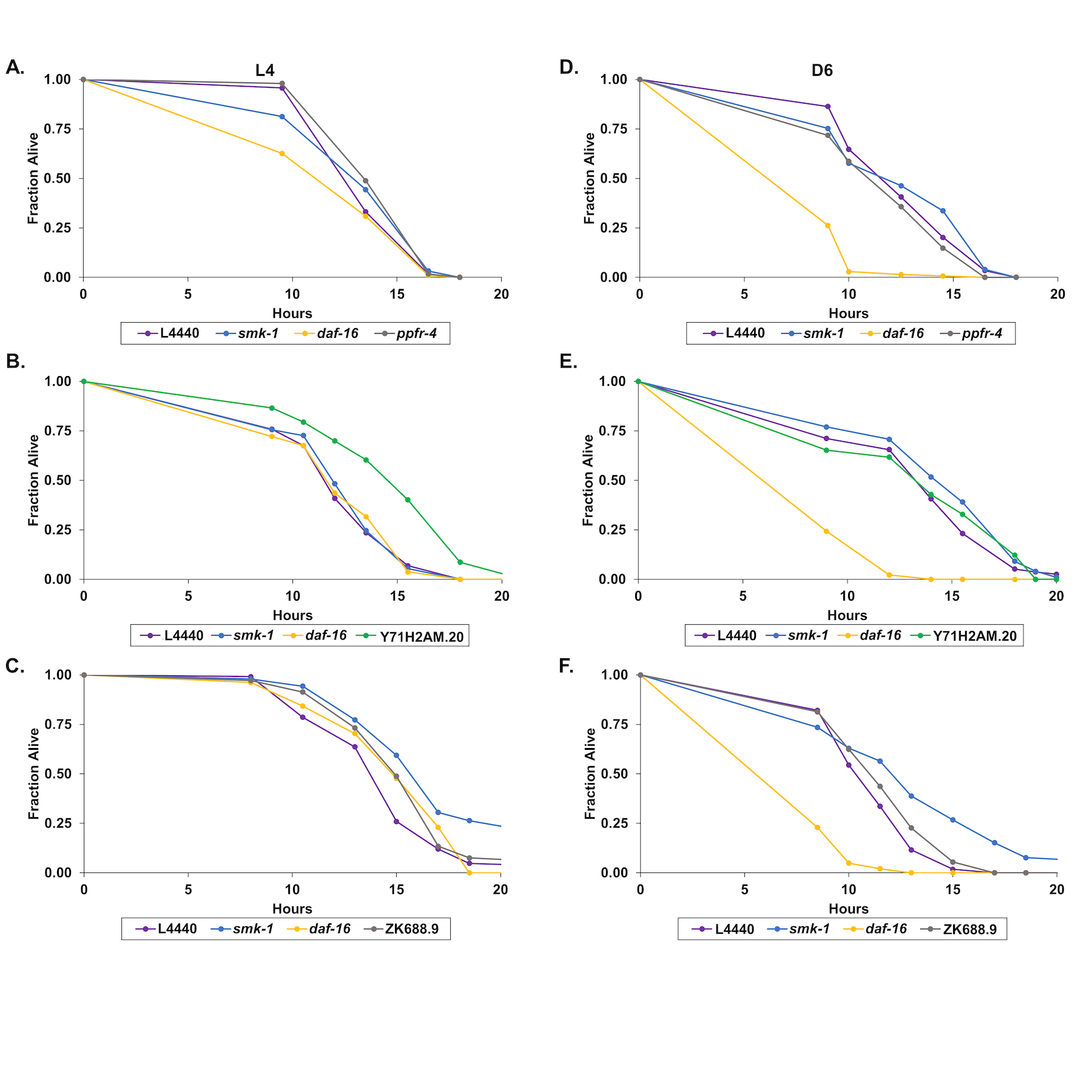

Supplement: S17 Fig — Beginning at the L1 stage the C. elegans homologs of human ⍺4 (ppfr-4; A, D), PTPA (Y71H2AM.20; B, E), and TIPRL (ZK688.9; C, F) were targeted with RNAi. Worms were shifted from 20˚ C to 35˚ C at larval stage L4 (A-C) or D6 (D-F) and maintained at the high temperature until death. A representative plot of the fraction of worms alive at each time point during the incubation at 35˚ C is shown. In all cases RNAi targeting daf-16 or smk-1 and the empty RNAi vector L4440 were included as controls. Statistical analyses indicate that RNAi treatments targeting ppfr-4, Y71H2AM.20, and ZK688.9 had no significant effect on the survival of worms under heat stress. (TIF) [file pone.0229812.s017.tif]
